# "Rebuilding Myself"- An intervention enhancing adaptability of cancer patients to return to work: a feasibility study

**DOI:** 10.1590/1980-220X-REEUSP-2024-0181en

**Published:** 2025-05-12

**Authors:** Yue Shi, Yujie Guo, Jingjing Dai, Jianli Lu, Suyun Huang, Xiaopeng Lu

**Affiliations:** 1 Nantong University, School of Nursing and Rehabilitation, Nantong, China.; 2 Nantong University, Affiliated Hospital, Nantong, China.; 3 Nantong University, Affiliated Maternity and Child Health Care Hospital, Nantong, China.; 4 Yangzhou University, Affiliated Taizhou Second People’s Hospital, Taizhou, China.; 5 Nantong Sixth People’s Hospital, Nantong, China.

**Keywords:** Cancer, Return to Work, Compliance, Nursing, Feasibility Studies, Câncer, Retorno ao Trabalho, Adaptabilidade, Enfermagem, Estudos de Viabilidade

## Abstract

**Objective:**

The aim of this research was to examine the feasibility and effects of the “Rebuilding Myself” intervention to enhance adaptability of cancer patients to return to work.

**Methods:**

A randomized controlled trial with a two-arm, single-blind design was employed. The control group received usual care, whereas the intervention group received “Rebuilding Myself” interventions. The effects were evaluated before the intervention, mid-intervention, and post-intervention. The outcomes were the adaptability to return to work, self-efficacy of returning to work, mental resilience, quality of life, and work ability.

**Results:**

The results showed a recruitment rate of 73.17%, a retention rate of 80%. Statistically significant differences were found between the two groups in cancer patients’ adaptability to return to work, self-efficacy to return to work, mental resilience, and the dimension of bodily function, emotional function, fatigue, insomnia, and general health of quality of life.

**Conclusion:**

“Rebuilding Myself” intervention was proven to be feasible and can initially improve cancer patients’ adaptability to return to work. The intervention will help provide a new direction for clinicians and cancer patients to return to work.

## RESUMO

**Objetivo:**

O objetivo desta pesquisa foi examinar a viabilidade e os efeitos da intervenção “Rebuilding Myself” para melhorar a adaptabilidade dos pacientes com câncer para retornar ao trabalho.

**Métodos:**

Um ensaio clínico controlado randomizado com um desenho de braço duplo e cegamento único foi empregado. O grupo de controle recebeu os cuidados habituais, enquanto o grupo intervenção recebeu a intervenção “Rebuilding Myself”. Os efeitos foram avaliados antes da intervenção, no meio da intervenção e após a intervenção. Os resultados foram a adaptabilidade para retornar ao trabalho, a autoeficácia para retornar ao trabalho, a resiliência mental, a qualidade de vida e a capacidade de trabalho.

**Resultados:**

Os resultados mostraram uma taxa de recrutamento de 73,17%, e uma taxa de retenção de 80%. Diferenças estatisticamente significativas foram encontradas entre os dois grupos nos pacientes com câncer em relação à adaptabilidade para retornar ao trabalho, a autoeficácia para retornar ao trabalho, a resiliência mental e a dimensão das funções corporais, funções emocionais, fadiga, insônia e saúde geral da qualidade de vida.

**Conclusão:**

A intervenção “Rebuilding Myself” mostrou ser viável e pode inicialmente melhorar a adaptabilidade dos pacientes com câncer para retornar ao trabalho. A intervenção ajudará a fornecer uma nova direção para clínicos e pacientes com câncer retornarem ao trabalho.

## INTRODUCTION

Cancer has emerged as a major public health issue worldwide^(1)^. Studies has been showed that around 40% of newly diagnosed cancer cases occur in adults within the working age range of 20–64 years^(2)^, with many of them being diagnosed while at work and subsequently taking leave for treatment. Individuals who have survived cancer and are still working have a significant impact on their capacity to retain and advance in their jobs due to the prolonged period of treatment and its adverse effects^(3)^. With advancements in diagnostic and treatment techniques, the five-year survival rates for cancer patients have increased^(4)^, leading to a steady rise in the number of individuals in recovery. According to the 2020 projections from the International Agency for Research on Cancer (IARC), the global population of cancer survivors who have lived for at least five years after diagnosis is expected to reach 50.6 million^(5)^. Consequently, the population of cancer survivors within the working-age group is also growing.

One of the key challenges for cancer patients after treatment is returning to work^(6)^. This refers to resuming full-time or part-time paid employment, including self-employment, either in the original or an adjusted position, with an average weekly work hour count^(7)^. Returning to work, as an important symbol of cancer patients’ disease outcome and reintegration into society, can reduce cancer survivors’ economic burden^(8)^; cancer survivors who return to work tend to exhibit better psychological resilience compared to those who do not rejoin the workforce. This reintegration helps them feel more connected to society^(9)^, fosters a sense of “normalcy” and boosts their self-esteem^(10)^, ultimately improving their overall quality of life^(11)^. Additionally, returning to work brings several societal benefits, such as higher workforce engagement, reduced social dependency, and smoother social development. Hence, the act of returning to work is crucial not only for the individual patient but also for the broader society.

In contrast, the current situation for cancer patients reentering the workforce in China is currently disappointing^(12)^. When it comes to resuming work, the majority of cancer survivors face physical, psychological, and social challenges. According to research, long-term treatment of the disease and its various side effects can cause survivors to suffer from issues such as body image disorder, low physical strength, and reduced work ability^(13)^; self-perceived shame, anxiety, depression, and other negative psychology also make it difficult for them to return to work^(14)^. Furthermore, social factors such as social alienation, occupational discrimination, and a lack of support from coworkers and leaders can result in unemployment, delayed employment, and impediments to promotion^(15)^. Despite the fact that the majority of cancer patients have side effects from the diagnosis and treatment^(16)^, many are eager to return to work, with the exception of those who are disabled^(17)^. Cancer adaptation, or psychosocial adjustment, refers to how an individual copes with the numerous complex challenges faced during cancer survival^(18)^. This adaptation is considered a key measure of a cancer patient’s overall health and quality of life, playing a crucial role in their psychosocial recovery. Patients find it difficult to adjust to returning to work after treatment due to a variety of variables such as national conditions, individual circumstances, and societal influences. These reasons include fear of disease recurrence, disease stigma, reluctance to return to work, low self-efficacy, and insufficient professional assistance at all levels^(19)^. Current evidence^(20)^ shows that China lacks intervention trials designed to support cancer patients in adapting to a return to work. In response, the research team developed the “Rebuilding Myself” intervention to evaluate its feasibility and effectiveness in enhancing cancer patients’ return-to-work adaptability. It was hypothesized that the intervention will boost patients’ ability to return to work, enhance their quality of life, and ultimately contribute to their full recovery.

## METHOD

### Study Design and Setting

**Figure 1:**
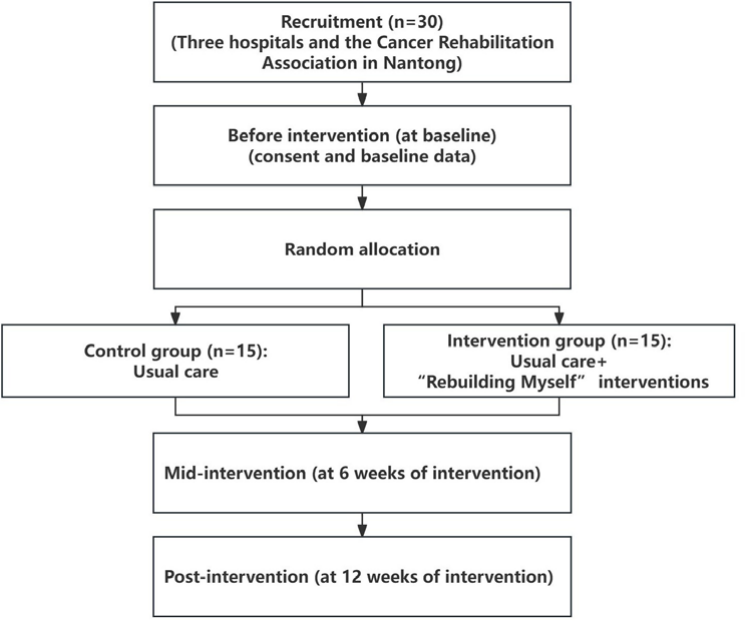
The study flowchart.

This study was designed as a two-arm, single-blind randomized controlled trial. The intervention protocol was developed based on the principles of creating and evaluating complex intervention strategies^(21)^ and followed a sequence of steps including formative research, feasibility assessment, pilot testing, and the randomized controlled trial. The feasibility study took place at the oncology outpatient departments and wards of three Grade 3, Class A hospitals, as well as the Cancer Rehabilitation Association in Nantong. The intervention spanned a three-month period and the research adhered to the CONSORT guidelines. A flowchart of the study can be seen in Figure 1.

### Participants

In this study, participants were screened through face-to-face interactions and recommendations from medical staff, and convenience sampling strategy was utilized to recruit participants who met the eligibility criteria. This study involved patients who met the following criteria: (1) had received a diagnosis of cancer; (2) were undergoing routine treatment in the hospital with no disease progression; (3) had previously been employed but had not returned to work at the time of the study; (4) were between 18 and 59 years old (as 60 is the retirement age in China); (5) were capable of reading, writing, and comprehending information; and (6) were aware of their diagnosis.

Patients were not included in the study if they had cognitive or psychiatric disorders, experienced other serious concurrent health conditions, or were participating in any other related studies.

Stopping criteria: Patients who experience disease recurrence, metastasis, or worsening during the intervention were eliminated. For patients who discontinue intervention, research groups took the following measures: 1. Provide psychological counseling to patients whose condition deteriorate in order to lessen their despair, grief, and fear while remaining optimistic; 2. Encourage patients to seek medical assessment and treatment as soon as possible, and conduct regular reviews; 3. Reduce stress by providing them with favorite activities such as playing music, viewing videos, communicating more frequently, and so on; 4. Attend to caregivers’ needs, encourage caregivers to discuss stressors, and offer positive assistance and support.

### Sample Size

At present, there is no uniform conclusion on the calculation method of the sample size of the pilot experiment by scholars at home and abroad. Previous studies have shown that a pilot experiment sample size of 30 cases is appropriate^(22)^. Therefore, 30 eligible cancer patients were included in this study, with 15 cases in each of the intervention and control groups.

### Randomization

Due to the small sample size allocated to each center in this study, the implementation of randomization in sub-centers was prone to imbalance, so the grouping method of central randomization was adopted^(23)^. Affected by the number of patients admitted to each center and the amount of surgery each year, it was difficult to achieve synchronization in continuous enrollment, so the method of competitive enrollment was adopted to include patients^(24)^. The number of enrolled patients in each center was not specified in advance, and the inclusion and deadline of research objects were unified. Each center began to recruit and screen patients at the same time to compete for inclusion. Randomization was carried out using computer-generated random numbers to create a random number table. After completing the baseline assessment, participants were randomly assigned to either the intervention group or the control group in a 1:1 ratio. Enrollment continued until the target number of patients was reached. In this study, an undergraduate medical student assigned numbers to the patients and used the ‘RANDBETWEEN’ function in Microsoft Excel to generate a set of random numbers. These numbers were then sorted in ascending order. The first half of the numbers were assigned a ‘1’ and placed in the intervention group, while the second half were assigned a ‘2’ and placed in the control group. The student wrote the numbers on sticky notes, which were then placed in sealed, opaque envelopes with sequential coding for secure storage. This study was conducted by a nursing graduate student who did not participate in data collection and analysis or disclose randomization.

### Blinding

In most cases, it was obvious to the patients whether they were in the intervention group or the control group, making it impossible to blind them to their group assignment. However, the randomization process described earlier ensured that the allocation sequence remained hidden from the researchers involved in patient recruitment, data collection, and analysis.

## INTERVENTIONS

### Theoretical Framework

Research has indicated that interventions without a theoretical framework tend to suffer from reduced quality and feasibility^(25)^. This protocol was grounded in a prior model^(26)^, which offered evidence to support the intervention approach.

**Figure 2:**
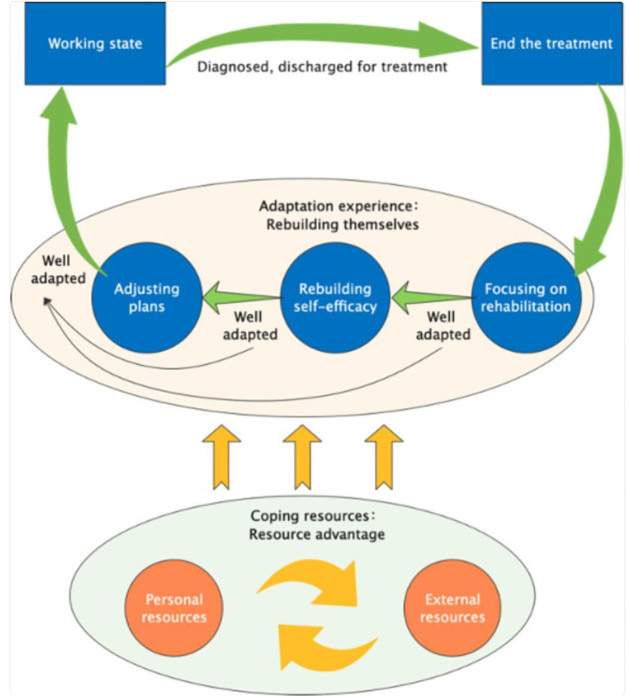
Cancer patients’ return-to-work adaptation experience and coping resources (reproduced with permission of author JS. X).

In the initial phase, the research team conducted in-depth interviews with 30 cancer patients who had successfully returned to work, using the grounded theory approach. From this, they developed the “Adaptation Experience and Coping Resource Model for cancer patients returning to work”^(26)^ (Figure 2). The model highlighted that the process of adapting to returning to work for cancer patients involved rebuilding oneself by leveraging available resources. The adaptation process was divided into three stages: focusing on rehabilitation, rebuilding self-efficacy, and adjusting and planning. The central concept of this model is the reconstruction of self.

The research team observed that these three stages were present throughout the entire rehabilitation process for the patients. The first stage, focusing on rehabilitation, serves as the starting point for returning to work, which includes healing, self-reflection, adjustment, and enhancing learning. Rebuilding self-efficacy is crucial for a successful return to work, involving accepting role models, receiving emotional support, and boosting confidence. The final stage, adjusting and planning, ensures that patients can successfully adapt to their work environment by seeking assistance and setting career plans. Coping resources are categorized into personal and external resources.

Therefore, based on the above theoretical model, research groups conducted a literature review and group discussion to design and form a draft intervention protocol; selected stakeholders to conduct structured interviews to collect their opinions and revise the draft; and used the Delphi method to select experts in the relevant fields to conduct an expert consultation, and based on their opinions, revised and finalized the “Rebuilding Myself” intervention protocol.

### Intervention Group

Prior to the implementation of the intervention, the research team was established, consisting of a nursing professor specializing in tumor psychosocial rehabilitation research, who oversaw team training, coordination, task distribution, and quality control. A psychological counselor was available to provide counseling to patients when necessary. Four nursing graduate students were responsible for data collection, patient recruitment, and intervention execution, while two medical undergraduate students handled the generation of allocation sequences and data analysis.

Patients in the experimental group participated in the “Rebuilding Myself” intervention, which consisted of three key themes and 16 courses delivered either in-person or online. The first goal was to improve the patients’ health management skills. This involved providing health education materials to help patients learn about cancer rehabilitation, guiding them to reflect on health risks, explore potential solutions, and support them in creating and following rehabilitation plans tailored to medical advice. The second focus was on rebuilding self-efficacy. Patients were encouraged to share their thoughts on returning to work, articulate their positive experiences, set achievable goals, and maintain a diary to track progress, all while strengthening their belief in recovery. It was crucial to gain insight into how the patient’s family, colleagues, supervisors, doctors, and nurses perceived the illness and the process of returning to work, in order to gather support from these individuals. To inspire patients, friends who had successfully overcome similar challenges were invited to share their return-to-work stories. Patients were also encouraged to modify their work-rest schedules and gradually resume work tasks to rebuild their confidence. The third step involved collaborating with medical professionals to create a personalized, step-by-step plan for returning to work and advancing in their careers, considering the patient’s recovery status and family and workplace circumstances. The objective was to create a balance between health and work, while also promoting the idea for patients to seek assistance from their family and workplace leaders.

This study applied an intervention that combined both face-to-face (one-on-one) and online (WeChat) methods. For the face-to-face component, the provider and patient collaboratively selected a quiet space for their discussions. The intervention intensity was tailored based on the patient’s individual circumstances and needs. Various methods were used, such as one-on-one communication interviews, family meetings, diary writing, and group sessions in small classes. Throughout the intervention, the researchers maintained contact with patients via phone or WeChat to encourage their continuous participation. The intervention format was based on the preferences of the patients, with the intensity of the intervention adjusted according to their conditions. Additionally, researchers continually assessed the patients’ physical and mental health, allowing for flexible adjustments to the intervention content and offering psychological counseling when needed. The specific details of the intervention are outlined below (Chart 1).

**Chart 1:** The detailed information of the intervention.

Themes	Objectives	Contents	Methods
Focus on rehabilitation	1. Understand the importance of returning to work. 2. Master the knowledge of physical and mental rehabilitation and self-management methods. 3. Implement the self-management plan.	1. Encourage patients to express their views on returning to work, communicate with patients about the positive significance of returning to work, and help them build their belief in comprehensive recovery. 2. Carry out health education actively, and give out health education leaflets. Encourage patients to strengthen their study, master the knowledge of cancer recovery and keep a good attitude. 3. Guide patients to reflect on factors that are detrimental to their physical and mental recovery (such as bad living habits, environmental factors, personality defects, et al.), discuss targeted solutions with patients, and seek support from peers, family members, and medical staff when necessary. 4. Ask patients’ healthcare providers about their health status and help them develop a self-health management plan. 5. Sign rehabilitation contracts with patients to enhance their compliance with health management. 1. Understand the views of the patient’s family members, peers, leaders, colleagues, and medical staff on patients’ illness and their return to work, and help them establish a correct view of rehabilitation. 2. Inform the patient’s family members, peers, leaders, colleagues, and medical staff of the importance of their care and support for their returning to work and complete recovery, and encourage them to offer their support. 3. Understand the condition of patients’ discussions with their family members, peers, colleagues, leaders, and medical staff about returning to work, ask them about their concerns and confusion on returning to work, and discuss solutions with patients. 4. Encourage patients to perceive the support from their own beliefs, family members, leaders, colleagues, peers, medical workers, and other aspects, record it in the diaries and review it regularly to constantly firm their belief of comprehensive recovery.	① ② ①③
Rebuild self-efficacy	Be familiar with ways to improve self-efficacy.	5. Guide patients to find examples of ‘role models’ who have successfully returned to work after cancer, and share the experience and positive energy gained. 6. Encourage patients to share their experiences of overcoming difficulties and achieving success in the past and the insights gained from them. 7. Self-confidence training ① Positive psychological suggestion training: urge patients to smile to themselves every day, repeat positive words, and give patients timely affirmation and praise. ② Mental resilience training: teach patients common stress coping skills, encourage patients to face pressure actively, and guide them to solve problems by clarifying and understanding issues, breaking complex problems into small steps, proposing solutions, and summarizing issues. ③ Patients are encouraged to gradually adjust their daily work and rest, and gradually integrate with the daily work and rest of the working stage. ④ Encourage the patient to do things related to work gradually. 1. Invite medical staff to make scientific decisions on the appropriate time, position, and workload for patients to return to work according to their conditions.	④ ⑤
Adjust and plan	Achieve the goal of returning to work gradually.	2. Based on the advice of the medical staffs, ask patients about communication with their family members, peers, leaders, colleagues, etc., and urge the patient to actively seek support for returning to work if necessary. 3. Ask the patient about his/her work goal, discuss with him/her appropriate career goals according to his/her recovery condition, make a gradual career plan, and evaluate the relationship between health and work. 4. Summarize the contents of this intervention protocol to enhance the adaptability of returning to work.	①

Methods: ①Individual communication and interview; ②Family meetings; ③Write diaries ; ④Thematical communication; ⑤Mini-classes.

### Control Group

The control group patients only received usual care, while researchers communicated personalized follow-up instructions and healing precautions to the patients via WeChat. Additionally, patients had the opportunity to ask the research team questions regarding rehabilitation through WeChat, and the team provided detailed and patient responses. This approach allowed the research team to maintain ongoing communication with patients, fostering trust and reducing patient dropout rates. In contrast, patients in the intervention group received the same standard care, along with the “Rebuilding Myself” intervention.

## ASSESSMENT OF THE OUTCOMES

### Sociodemographic Data

The following sociodemographic data were measured in the form of questionnaires at baseline: gender, age, marital status (married, single, widowed, divorced), education (primary school or below, junior high school or technical secondary school, senior high school or junior college, undergraduate, master degree or above), place of residence (rural, town, city), religious beliefs (yes, no), occupation before illness, medical insurance, cancer diagnosis and stage, treatment (surgery, radiotherapy, chemotherapy, other).

### Feasibility Metrics

Feasibility metrics in this study include recruitment rates, retention rates and intervention satisfaction. The specific calculation method is as follows:

(1) Recruitment rates: the number of patients who agree to participate in the study as a percentage of the number of patients who meet the inclusion and exclusion criteria.
(2) Retention rates: the number of patients who complete the entire intervention and filled out the questionnaire as a percentage of the number of patients measured at baseline before the intervention.
(3) Intervention satisfaction: The Likert 5-level scoring method is used, which is divided into five levels: very dissatisfied, dissatisfied, average, satisfied, and very satisfied, with scores ranging from 1 to 5 respectively. After the intervention, the patients evaluated the entire intervention process.

### Outcome Measures

The main outcome measure was the cancer patients’ adaptability to returning to work^(27)^. The scale used to assess this was developed by our research team and has demonstrated strong reliability and validity, with a Cronbach’s α coefficient of 0.973. It consists of 24 items divided into three categories: rehabilitation focus (6 items), rebuilding self-efficacy (9 items), and adjustment and planning (9 items). The scale employs a 5-point Likert scale, ranging from “strongly disagree” to “strongly agree”, with scores assigned from 1 to 5. A higher overall score reflects greater adaptability in patients’ ability to return to work.

The secondary outcome measures were self-efficacy of returning to work, mental resilience, quality of life and work ability.

The Chinese version of the Return-to-Work Self-Efficacy Questionnaire^(28)^ assesses patients’ self-efficacy in their ability to return to work. The Cronbach’s α coefficient for this scale ranges between 0.90 and 0.96, indicating strong reliability. The questionnaire consists of 11 items, with items 2, 6, and 9 requiring reverse scoring. It utilizes a 6-point Likert scale, where responses range from 1 to 6 points. The overall score is calculated as the average of the 11 items, with a score above 4.5 indicating that the patient has a higher level of self-efficacy regarding returning to work.

The Connor-Davidson Resilience Scale^(29)^ was used to assess the mental resilience of cancer patients. It was modified by Chinese researchers into three dimensions: tenacity (13 items), strength (8 items), and optimism (4 items). The scale has a Cronbach’s α coefficient of 0.89, with a test-retest reliability of 0.87. Each item is rated on a scale from 0 to 5, where 0 means “never” and 5 means “always”. A higher total score indicates greater psychological flexibility.

The Chinese version of the European Organization for Research and Treatment of Cancer Quality of Life Questionnaire-Core 30^(30)^ was used to assess the quality of life of cancer patients. This scale consists of 30 items, organized into 15 dimensions: 5 functional dimensions (physical, role, cognitive, emotional, and social function), 3 symptom dimensions (fatigue, pain, nausea and vomiting), 1 dimension for general health/quality of life, and 6 individual items (shortness of breath, insomnia, loss of appetite, constipation, diarrhea, and financial difficulties). The Cronbach’s α coefficient for each dimension of the Chinese EORTC QLQ-C30 exceeds 0.7, except for cognitive function, and the test-retest reliability is above 0.73. Items 29 and 30 are scored on a 7-point scale, ranging from “very poor” to “very good” (1 to 7 points), while the remaining items use a 4-point scale (1 to 4 points). Higher scores in the functional dimensions and general health status indicate better quality of life, while higher scores in the symptom dimensions reflect worse quality of life for cancer patients.

The Chinese version of the Work Ability Index (WAI) questionnaire^(31)^ was used to assess an individual’s capability to perform their job. This scale evaluates both the physical and mental demands of various roles, as well as the person’s health and mental resources. It consists of 7 components: work ability assessment, needs, forecasts, work impact, illness, absenteeism, and psychological condition, with a maximum score of 49 points. The Cronbach’s α coefficients for all the subscales exceed 0.70, indicating satisfactory reliability^(32)^. A higher score on the questionnaire reflects a greater ability to perform job-related tasks.

## DATA COLLECTION AND ANALYSIS

Data to assess the intervention were gathered at three time points: at baseline, the 6th week, and after the 12th week, using a self-completion questionnaire. An investigator, who was not involved in the implementation or analysis of the intervention, collected the data. Statistical analyses were performed using SPSS version 26.0, with a significance level set at α = 0.05. Descriptive statistics, such as percentages, means, and standard deviations, were used to summarize sociodemographic and outcome variables. To compare baseline characteristics and outcome measures between the two groups, T-tests or the Mann-Whitney U test for continuous variables, and chi-square tests for categorical variables were employed. Additionally, both per-protocol (PP) and intention-to-treat (ITT) analyses were conducted to assess the intervention’s effectiveness, accounting for potential patient dropouts and exclusions. In cases of missing data, values from previous measurements were carried forward to replace missing subsequent data. Since ITT analysis tends to underestimate the intervention’s effect, and PP analysis may overestimate it, the reliability of the study is enhanced when both approaches yield similar results^(33)^. Consequently, both analysis methods were applied in this study to evaluate the intervention outcomes.

## ETHICAL CONSIDERATIONS

This research received ethical approval from the Ethics Committee of Nantong University on February 15, 2019 (Approval No. (2019)15) and was registered with the Chinese Clinical Trial Registry on March 23, 2022, under the registration number ChiCTR2200057943.

## RESULTS

### Feasibility Analysis Results

The researcher recruited 41 cancer patients who met the inclusion exclusion criteria, and 30 agreed to participate in the study and completed the general information questionnaire and baseline assessment questionnaire, which calculated a recruitment rate of 73.17%. During the implementation of the intervention, 4 (13.33%) patients were excluded due to disease recurrence, metastasis, and deterioration of the disease and readmitted to the hospital for treatment, and 2 patients (6.67%) withdrew due to a change in their willingness to return to work. Thus, a total of 24 patients in both groups completed the entire intervention and questionnaire completion, which was calculated to give a retention rate of 80%. Upon completion of the intervention, 3 patients in the intervention group felt “fair”, 4 “satisfied”, and 8 “very satisfied”; 9 patients in the control group felt “general”, 5 “satisfied”, 1 “very satisfied”, the two groups of patients intervention satisfaction scores for the chi-square test, standardized test statistical value of 2.781, P = 0.008 (P < 0.05), have statistical significance, indicating that patient satisfaction in the intervention group was significantly higher than that in the control group.

## EFFECTIVENESS EVALUATION RESULTS

### Comparison of Baseline Data

The marital status of the patients in both groups was married, and none of them had any religious beliefs; the rest of the general conditions were analyzed by the chi-square test, and the results showed that the P-value was greater than 0.05, and they were comparable (Results are detailed in Table 1).

**Table 1:** Comparison of the general conditions of the two groups of patients-Nantong, China, 2022.

	Themes	Control Group (N = 15)	Intervention group (N = 15)	*χ* ^2^	P
	≤40	3	3		
Age	41~50	7	9	0.750	0.687
	>50	5	3		
Gender	Male	2	2	0.000	1.000
	Female	13	13		
	Primary education or no diploma	1	1		
	Junior high school	7	7		
Educational level	High school or technical secondary school	4	6	4.400	0.355
	Junior college	3	0		
	Bachelor degree or above	0	1		
	Countryside	2	2		
Place of residence	Town	6	10	2.600	0.273
	City	7	3		
	New rural medical insurance	1	1		
Medical insurance	Urban resident medical insurance	6	9	1.292	0.524
	Employee medical insurance	8	5		
	Breast cancer	8	10		
	Cervical cancer	2	0		
	Thyroid cancer	1	0		
	Thymic cancer	1	1		
Cancer type	Lung cancer	1	1	6.222	0.514
	Liver cancer	1	0		
	Lymphoma	0	2		
	Colon cancer	1	1		
	Worker	5	6		
	Staff	3	2		
	Professional skill worker	2	1		
Type of employment	Business and service personnel	2	1	2.958	0.707
	Government agency personnel	0	2		
	Other	3	3		
	I	4	2		
Disease Stage	II	8	8	1.167	0.191
	III	3	1		
	Only surgical treatment	3	1		
Treatments	Radiation, chemotherapy, targeted therapy, or immunotherapy only	2	4	1.667	0.435
	Combining multiple treatment modalities	10	10		

Métodos: ①Comunicación individual y entrevista; ②Reunión de família; ③Escribir diários; ④Comunicación temática; ⑤Clases.

### Comparison of Effectiveness Evaluation Index Scores Between the Two Groups at Baseline

The baseline analysis of the effectiveness evaluation indicators for both patient groups revealed a P value greater than 0.05, which was comparable (for more details, refer to Table 2).

**Table 2:** Comparison of effectiveness evaluation index scores before intervention (baseline) between the two groups-Nantong, China, 2022.

Evaluation index		Control group	Intervention group	Statistics	*P*
Adaptability to return to work ( $\bar{x}$ ± s)		85.47 ± 7.62	85.87 ± 8.66	0.134	0.894
Return to work self-efficacy ( $\bar{x}$ ± s)		4.09 ± 0.50	4.09 ± 0.46	–0.034	0.973
Mental resilience ( $\bar{x}$ ± s)		65.00 ± 7.97	65.47 ± 7.78	–0.162	0.872
Ability to work ( $\bar{x}$ ± s)		28.63 ± 7.70	29.00 ± 6.15	–0.144	0.887
Quality of Life [M(P_25_,P_75_)]	Bodily function	86.67(83.33, 93.33)	86.67(86.67, 93.33)	–0.086	0.931
	Role function	83.33(75.00, 100.00)	83.33(75.00, 91.67)	–0.396	0.692
	Emotional function	83.33(70.83, 91.67)	83.33(75.00, 83.33)	–0.774	0.439
	Cognitive function	83.33(83.33, 100.00)	83.33(83.33, 100.00)	–0.268	0.789
	Social function	66.67(66.67, 83.33)	83.33(66.67, 83.33)	–0.746	0.456
	Fatigue	77.78(66.67, 88.89)	77.78(66.67, 83.33)	–0.087	0.931
	Pain	100.00(100.00, 100.00)	100.00(91.67, 100.00)	–0.505	0.614
	Shortness of breath	100.00(100.00, 100.00)	100.00(100.00, 100.00)	0.000	1.000
	Insomnia	66.67(50.00, 83.33)	66.67(33.33, 100.00)	–0.242	0.809
	Loss of appetite	100.00(100.00, 100.00)	100.00(100.00, 100.00)	–0.544	0.586
	Feel sick and vomit	100.00(100.00, 100.00)	100.00(100.00, 100.00)	–0.949	0.343
	Constipate	100.00(100.00, 100.00)	100.00(100.00, 100.00)	–0.598	0.550
	Diarrhea	100.00(100.00, 100.00)	100.00(100.00, 100.00)	0.000	1.000
	Economic difficulties	100.00(66.67, 100.00)	100.00(66.67, 100.00)	–1.282	0.200
	General health	66.67(62.50, 70.83)	66.67(66.67, 66.67)	–0.345	0.730

### Comparison of Effectiveness Evaluation Index Scores Between the Two Groups in the Middle Period (6th Week) and Late (12th Week)

The study findings indicated that in the middle stage of the intervention, specifically during the 6th week, the PP analysis results revealed no statistically significant differences in the scores of the assessment tools between the two patient groups (P > 0.05) (refer to Table 3 for details).

**Table 3:** Comparison of effectiveness evaluation index scores between the two groups at mid-intervention (at 6 weeks of intervention) (PP analysis) - Nantong, China, 2022.

Evaluation index		Control group	Intervention group	Statistics	*P*
Adaptability to return to work ( $\bar{x}$ ± s)		86.07 ± 7.81	93.27 ± 9.38	2.048	0.054
Return to work self-efficacy ( $\bar{x}$ ± s)		4.16 ± 0.47	4.50 ± 0.47	–1.813	0.084
Mental resilience ( $\bar{x}$ ± s)		65.14 ± 8.48	71.36 ± 7.83	1.902	0.070
Ability to work ( $\bar{x}$ ± s)		29.21 ± 7.10	32.27 ± 5.13	–1.250	0.224
Quality of Life [M(P_25_,P_75_)]	Bodily function	90.00(60.00, 100.00)	93.33(73.33, 100.00)	–1.042	0.298
	Role function	83.33(50.00, 100.00)	83.33(66.67, 100.00)	–0.120	0.904
	Emotional function	83.33(66.67, 100.00)	83.33(75.00, 100.00)	–0.403	0.687
	Cognitive function	83.33(66.67, 100.00)	100.00(66.67, 100.00)	–0.458	0.647
	Social function	83.33(66.67, 100.00)	83.33(66.67, 100.00)	–0.522	0.602
	Fatigue	77.78(55.56, 100.00)	77.78(66.67, 100.00)	–1.299	0.194
	Pain	100.00(83.33, 100.00)	100.00(66.67, 100.00)	–0.233	0.816
	Shortness of breath	100.00(66.67, 100.00)	100.00(66.67, 100.00)	–0.258	0.796
	Insomnia	66.67(33.33, 100.00)	66.67(33.33, 100.00)	–0.694	0.488
	Loss of appetite	100.00(66.67, 100.00)	100.00(66.67, 100.00)	–0.389	0.697
	Feel sick and vomit	100.00(100.00, 100.00)	100.00(83.33, 100.00)	–1.128	0.259
	Constipate	100.00(66.67, 100.00)	100.00(66.67, 100.00)	–0.389	0.697
	Diarrhea	100.00(100.00, 100.00)	100.00(100.00, 100.00)	0.000	1.000
	Economic difficulties	100.00(33.33, 100.00)	100.00(0.00, 100.00)	–0.841	0.401
	General health	66.67(50.00, 83.33)	75.00(66.67, 83.33)	–1.685	0.092

The study results demonstrated that at the 6th week of the intervention, which marks the middle stage, the ITT analysis indicated no statistically significant differences in the scores across the assessment tools between the two patient groups (P > 0.05) (see Table 4 for further details).

**Table 4:** Comparison of effectiveness evaluation index scores between the two groups at mid-intervention (at 6 weeks of intervention) (ITT analysis)-Nantong, China, 2022.

Evaluation index		Control group	Intervention group	Statistics	*P*
Adaptability to return to work ( $\bar{x}$ ± s)		85.67 ± 7.69	90.40 ± 10.23	1.433	0.164
Return to work self-efficacy ( $\bar{x}$ ± s)		4.10 ± 0.51	4.33 ± 0.53	–1.179	0.248
Mental resilience ( $\bar{x}$ ± s)		65.13 ± 8.17	69.80 ± 7.95	1.586	0.124
Ability to work ( $\bar{x}$ ± s)		28.37 ± 7.58	30.20 ± 7.75	–0.746	0.462
Quality of Life [M(P_25_,P_75_)]	Bodily function	86.67(60.00, 100.00)	93.33(73.33, 100.00)	–0.763	0.445
	Role function	83.33(50.00, 100.00)	83.33(66.67, 100.00)	–0.428	0.669
	Emotional function	83.33(66.67, 100.00)	83.33(75.00, 100.00)	–0.557	0.578
	Cognitive function	83.33(66.67, 100.00)	100.00(66.67, 100.00)	–1.105	0.269
	Social function	83.33(0.00, 100.00)	83.33(33.33, 100.00)	–0.677	0.498
	Fatigue	77.78(55.56, 100.00)	77.78(66.67, 100.00)	–1.590	0.112
	Pain	100.00(66.67, 100.00)	100.00(33.33, 100.00)	–0.105	0.916
	Shortness of breath	100.00(66.67, 100.00)	100.00(66.67, 100.00)	–0.482	0.630
	Insomnia	66.67(33.33, 100.00)	66.67(33.33, 100.00)	–1.060	0.289
	Loss of appetite	100.00(33.33, 100.00)	100.00(66.67, 100.00)	–1.089	0.276
	Feel sick and vomit	100.00(66.67, 100.00)	100.00(83.33, 100.00)	–0.518	0.605
	Constipate	100.00(66.67, 100.00)	100.00(66.67, 100.00)	–0.598	0.550
	Diarrhea	100.00(66.67, 100.00)	100.00(100.00, 100.00)	–1.000	0.317
	Economic difficulties	100.00(0.00, 100.00)	100.00(0.00, 100.00)	–1.282	0.200
	General health	66.67(50.00, 83.33)	66.67(66.67, 83.33)	–1.705	0.088

At the 12th week of the intervention, the PP analysis results revealed statistically significant differences between the two groups in several areas, including the adaptability of returning to work, self-efficacy in returning to work, mental resilience, and dimensions related to bodily function, emotional function, fatigue, insomnia, and general health of quality of life (P < 0.05). However, no significant differences were found in the scores for the other indexes and dimensions (P > 0.05) (refer to Table 5 for details).

**Table 5:** Comparison of effectiveness evaluation index scores between the two groups in the later stage of intervention (at 12 weeks of intervention) (PP analysis)-Nantong, China, 2022.

Evaluation index		Control group	Intervention group	Statistics	*P*
Adaptability to return to work ( $\bar{x}$ ± s)		86.14 ± 7.75	97.70 ± 7.79	3.592	0.002**
Return to work self-efficacy ( $\bar{x}$ ± s)		4.16 ± 0.47	4.72 ± 0.40	–3.131	0.005**
Mental resilience ( $\bar{x}$ ± s)		65.29 ± 8.27	73.90 ± 8.54	2.468	0.023*
Ability to work ( $\bar{x}$ ± s)		29.75 ± 6.67	35.00 ± 6.51	–1.927	0.068
Quality of Life [M(P_25_, P_75_)]	Bodily function	90.00(60.00, 100.00)	100.00(73.33, 100.00)	–2.300	0.021*
	Role function	91.67(66.67, 100.00)	91.67(83.33, 100.00)	–0.166	0.868
	Emotional function	83.33(66.67, 100.00)	91.67(83.33, 100.00)	–2.221	0.026*
	Cognitive function	83.33(66.67, 100.00)	100.00(83.33, 100.00)	–0.811	0.418
	Social function	83.33(66.67, 100.00)	83.33(66.67, 100.00)	–1.354	0.176
	Fatigue	83.33(66.67, 100.00)	88.89(77.78, 100.00)	–2.303	0.021*
	Pain	100.00(83.33, 100.00)	100.00(66.67, 100.00)	–0.244	0.807
	Shortness of breath	100.00(66.67, 100.00)	100.00(100.00, 100.00)	–0.845	0.398
	Insomnia	66.67(33.33, 100.00)	50.00(33.33, 100.00)	–2.482	0.013*
	Loss of appetite	100.00(66.67, 100.00)	100.00(66.67, 100.00)	–0.845	0.398
	Feel sick and vomit	100.00(100.00, 100.00)	100.00(83.33, 100.00)	0.259	1.000
	Constipate	100.00(66.67, 100.00)	100.00(66.67, 100.00)	–1.222	0.222
	Diarrhea	100.00(100.00, 100.00)	100.00(100.00, 100.00)	0.000	1.000
	Economic difficulties	100.00(33.33, 100.00)	100.00(0.00, 100.00)	–0.426	0.670
	General health	66.67(50.00, 83.33)	83.33(66.67, 83.33)	–3.669	0.000***

At the 12th week of the intervention, the ITT analysis revealed statistically significant differences between the two groups in terms of the adaptability of returning to work, as well as the dimensions of fatigue, insomnia, and general health within quality of life (P < 0.05). However, no statistically significant differences were observed for the remaining indicators and dimensions (P > 0.05) (see Table 6 for more details).

**Table 6:** Comparison of effectiveness evaluation index scores between the two groups in the later stage of intervention (at 12 weeks of intervention) (ITT analysis)-Nantong, China, 2022.

Evaluation index		Control group	Intervention group	Statistics	*P*
Adaptability to return to work ( $\bar{x}$ ± s)		85.73 ± 7.63	92.73 ± 10.46	2.094	0.046*
Return to work self-efficacy ( $\bar{x}$ ± s)		4.10 ± 0.51	4.46 ± 0.54	–1.860	0.073
Mental resilience ( $\bar{x}$ ± s)		65.27 ± 7.97	71.20 ± 8.65	1.953	0.061
Ability to work ( $\bar{x}$ ± s)		28.87 ± 7.28	32.00 ± 7.17	–1.187	0.245
Quality of Life [M(P_25_,P_75_)]	Bodily function	86.67(60.00, 100.00)	93.33(73.33, 100.00)	–1.373	0.170
	Role function	83.33(50.00, 100.00)	83.33(66.67, 100.00)	–0.115	0.909
	Emotional function	83.33(66.67, 100.00)	91.67(75.00, 100.00)	–1.651	0.099
	Cognitive function	83.33(66.67, 100.00)	100.00(83.33, 100.00)	–1.538	0.124
	Social function	83.33(0.00, 100.00)	83.33(33.33, 100.00)	–0.920	0.358
	Fatigue	77.78(55.56, 100.00)	77.78(66.67, 100.00)	–2.077	0.038*
	Pain	100.00(66.67, 100.00)	100.00(66.67, 100.00)	–0.598	0.550
	Shortness of breath	66.67(33.33, 100.00)	66.67(33.33, 100.00)	–2.323	0.020*
	Insomnia	100.00(33.33, 100.00)	100.00(66.67, 100.00)	–0.637	0.524
	Loss of appetite	100.00(66.67, 100.00)	100.00(83.33, 100.00)	–0.048	0.962
	Feel sick and vomit	100.00(66.67, 100.00)	100.00(66.67, 100.00)	–1.439	0.150
	Constipate	100.00(66.67, 100.00)	100.00(100.00, 100.00)	–1.000	0.317
	Diarrhea	100.00(0.00, 100.00)	100.00(0.00, 100.00)	–1.282	0.200
	Economic difficulties	66.67(50.00, 83.33)	66.67(66.67, 83.33)	–2.975	0.003**
	General health	77.78(55.56, 100.00)	77.78(66.67, 100.00)	–2.077	0.038*

PS:*P < 0.05

**P < 0.01

***P < 0.001

## DISCUSSION

The intervention protocol was based on the theoretical model constructed by our team, and through literature review, group discussion, structured interviews, and Delphi expert consultation, the protocol was constructed to fully understand the suggestions of different groups of people, such as cancer patients and their families, clinical staff, and experts in various fields. The intervention protocol took into account the individualized differences of cancer patients and adopted face-to-face (one-on-one) and online (WeChat) interventions that complement each other; at the same time, it developed personalized interventions based on the patients’ own conditions and needs without interfering with their daily lives or increasing their burden.

The results of feasibility study revealed an intervention recruitment rate of 73.17%, showing that the majority of cancer patients in remission had intervention-related requirements, were willing to participate in this intervention study, and were eager to adjust to returning to work. Studies have shown that medical staff recommendation is an effective method of recruitment^(34)^, and 21 patients (70%) were recruited using this method in this study, which promotes the development of a trusting relationship between the intervention implementer and the patient and improves intervention adherence. Second, the findings revealed that patients in the intervention group were substantially more satisfied with the intervention than the control group, indicating that cancer patients were willing to accept it. Furthermore, the intervention retention rate in this study was 80%, with 4 (13.33%) patients dropping out due to disease recurrence, metastasis, and worsening re-admission to the hospital for treatment, and 2 patients (6.67%) withdrawing from the study due to a change in their willingness to return to work, indicating that the participants’ adherence to the intervention in this study was good. In addition, the randomized controlled trial results indicated that the protocol can improve patients’ adaptability to return to work, self-efficacy to return to work, mental resilience, and the dimension of bodily function, emotional function, fatigue, insomnia, and general health of quality of life, which has shown preliminary effects and provides a practical basis for further large-sample clinical trials in the future. In summary, the intervention protocol is feasible.

A study^(35)^ highlighted that the return-to-work process for cancer patients is influenced by multiple factors and necessitates an integrated approach that combines psychological, vocational, and physical methods. In a Cochrane systematic review^(36)^, the authors categorized interventions aimed at improving cancer patients’ work return adaptability into four types: physical, psychological, occupational, and multidisciplinary, providing definitions for each category. This protocol incorporates physical, psychological, and occupational methods to offer multidisciplinary rehabilitation support to patients, with the goal of enhancing their ability to return to work as part of a comprehensive rehabilitation strategy. Thus, this research protocol demonstrates practical applicability.

This study conducted a randomized controlled trial to assess the adaptability of Chinese cancer patients in returning to work. From a theoretical innovation perspective, the research team’s earlier grounded theory study, based on credible sources, developed a theoretical model to understand cancer patients’ experiences and coping resources related to returning to work, and introduced the novel concept of “return to work adaptability”. The team also proposed the “Rebuilding Myself” adaptive intervention protocol for cancer patients, incorporating diverse intervention strategies. In terms of practical innovation, the study’s outcomes could motivate future clinical researchers to conduct large-scale, multi-center randomized controlled trials. The validated intervention protocols may assist cancer patients in resuming their work and achieving psychosocial recovery, providing a foundation for further research and new directions in the field of work return studies.

## LIMITATION

There were still some deficiencies in this research. The results of the feasibility study showed that the advantage of this intervention to improve patients’ work ability was not obvious, which might be related to the shorter intervention time of the study; the results of the PP and ITT analyses were partially inconsistent, and the credibility of the study results needs to be improved. In addition, the sample size included in the study was small, and the results were more unstable; in the future, the sample size can be further expanded and the number of follow-up visits can be increased to further explore the long-term effects of this protocol.

## CONCLUSION

This study introduced the “Rebuilding Myself” intervention for the physical and mental recovery of cancer patients for the first time. The intervention protocol demonstrated its feasibility and showed initial improvements in cancer patients’ adaptability to return to work, self-efficacy to return to work, mental resilience, and overall quality of life. The protocol was of great value to the gradual realization of comprehensive physical, psychological, and social rehabilitation of cancer patients. It also has the potential to guide the development of future randomized controlled trials and provide new ideas for patients wishing to return to work.

## References

[1] Henley SJ, Ward EM, Scott S, Ma J, Anderson RN, Firth AU (2020). Annual report to the nation on the status of cancer, part I:
national cancer statistics. Cancer.

[2] Kiasuwa Mbengi R, Otter R, Abatih E, Goetghebeur E, Bouland C, de Brouwer C (2018). Utilisation de l’échantillon permanent (eps) pour l’étude du
retour au travail après cancer. Défis et opportunités pour la recherche. Rev Med Brux..

[3] Tang J, Guo YJ, Que WQ (2022). Construction and preliminary validation of an intervention
protocol named ‘Rebuilding oneself’ to enhance caner patients’ adaptation to
return to work. Nurs J Chin PLA.

[4] Li Q, Xia C, Li H, Yan X, Yang F, Cao M (2024). Disparities in 36 cancers across 185 countries: secondary
analysis of global cancer statistics. Front Med.

[5] IARC (2020). Estimated number of prevalent cases in 2020, all cancers, both sexes,
all ages.

[6] Traversa P. (2021). 36th Annual CAPO Conference: Advocating for All: Psychosocial
Oncology at the Intersections of Equity, Diversity, and Inclusion, 8–10 June
2021. Curr Oncol.

[7] Li JM, Su XQ, Xu XP, Xue P, Guo YJ (2023). Influencing factors analysis of adaptability of cancer patients
to return-to-work. Support Care Cancer.

[8] Lim ZW, Wang CC, Wu WT, Chen WL (2022). Return to work in survivors with occupational
cancers. J Occup Environ Med.

[9] Xu W, Hu D, Chen H, Li N, Feng X, Hu M (2024). Quality of working life and adaptability of returning to work in
nurse cancer survivors: a cross-sectional study. Support Care Cancer.

[10] Butow P, Laidsaar-Powell R, Konings S, Lim CYS, Koczwara B (2020). Return to work after a cancer diagnosis: a meta-review of reviews
and a meta-synthesis of recent qualitative studies. J Cancer Surviv.

[11] Ruiz de Azua G, Kousignian I, Vaz-Luis I, Di Meglio A, Caumette E, Havas J (2023). Sustainable return to work among breast cancer
survivors. Cancer Med.

[12] Wang ML, Liu JE, Wang HY, Chen J, Li YY (2014). Posttraumatic growth and associated socio-demographic and
clinical factors in Chinese breast cancer survivors. Eur J Oncol Nurs.

[13] Xunlin NG, Lau Y, Klainin-Yobas P (2020). The effectiveness of mindfulness-based interventions among cancer
patients and survivors: a systematic review and
meta-analysis. Support Care Cancer.

[14] Chen YJ, Lai YH, Lee YH, Tsai KY, Chen MK, Hsieh MY (2021). Impact of illness perception, mental adjustment, and
sociodemographic characteristics on return to work in patients with head and
neck cancer. Support Care Cancer.

[15] Sheppard DM, Frost D, Jefford M, O’Connor M, Halkett G (2020). Building a novel occupational rehabilitation program to support
cancer survivors to return to health, wellness, and work in
Australia. J Cancer Surviv.

[16] Ketterl TG, Syrjala KL, Casillas J, Jacobs LA, Palmer SC, McCabe MS (2019). Lasting effects of cancer and its treatment on employment and
finances in adolescent and young adult cancer survivors. Cancer.

[17] de Boer AG, Tamminga SJ, Boschman JS, Hoving JL (2024). Non-medical interventions to enhance return to work for people
with cancer. Cochrane Database Syst Rev.

[18] Yao JJ, Chen RN, Liu YY, Yuan CR (2013). The level of psychosocial adjustment in cancer patients and its
influencing factors. Nurs J Chin..

[19] Li L, Fang F, Li H, Ma LY, Cui RZ (2022). Longitudinal qualitative study of continuous care needs in
gynecological malignancies survivors. Morden Nurs.

[20] Guo YJ, Tang J, Li JM, Zhu LL, Xu JS (2021). Exploration of interventions to enhance return-to-work for cancer
patients: A scoping review. Clin Rehabil.

[21] Polit DF, Beck CT (2016). Nursing research generating and assessing evidence for nursing
practice.

[22] Teresi JA, Yu X, Stewart AL, Hays RD (2022). Guidelines for designing and evaluating feasibility pilot
studies. Med Care.

[23] Krisam J, Ryeznik Y, Carter K, Kuznetsova O, Sverdlov O (2024). Understanding an impact of patient enrollment pattern on
predictability of central (unstratified) randomization in a multi-center
clinical trial. Stat Med.

[24] Design and Implementation of Randomized Controlled Trial of
Multicenter Nursing Intervention. (2024). A case study of breast cancer patients’ return to family
intervention [J]. China Nursing Management.

[25] Frances ML, Zhang NQ, Liu J, Yuwen WC (2016). Designing evidence-based interventions: from descriptive to
clinical trials research. Chin Nurs Manage.

[26] Xu J, Zhou Y, Li J, Tang J, Hu X, Chen Y (2023). Cancer patients’ return-to-work adaptation experience and coping
resources: a grounded theory study. BMC Nurs.

[27] Li JM, Guo YJ, Gu LP, Zhou XY, Xu JS, Hu XY (2021). Assessment scale for cancer patients’ adaptability of returning
to work: Development and Validation of Reliabilty &
Validity. Nurs J Chin PLA..

[28] Gao YX, Qu QR, Wang BX, Zhang KX, Cui TJ (2021). Chinese translation of the return-to-work self-efficacy
questionnaire in cancer patients and its reliability and validity
test. Nurs J Chin PLA..

[29] Yu X, Zhang JX (2007). Factor analysis and psychometric evaluation of the
Connor-Davidson Resilience Scale (CD-RISC) with Chinese
people. Soc Behav Personal.

[30] Wan CH (1999). The measurement and evaluation methods of quality of life.

[31] Ma LJ (2000). Work ability Index (WAI) questionnaire. Lab Med..

[32] Guo Y, Xie H, Ding L, Shi Y, Han P (2024). Effects of a ‘Rebuilding Myself’ intervention on enhancing the
adaptability of cancer patients to return to work: a randomized controlled
trial. BMC Cancer.

[33] Ahn E, Kang H (2023). Intention-to-treat versus as-treated versus per-protocol
approaches to analysis. Korean J Anesthesiol.

[34] Cochrane BB, Lewis FM, Griffith KA (2011). CExploring a diffusion of benefit: does a woman with breast
cancer derive benefit from an intervention delivered to her partner?
[J]. Oncol Nurs Forum.

[35] Thijs KM, de Boer AG, Vreugdenhil G, van de Wouw AJ, Houterman S, Schep G (2012). Rehabilitation using high-intensity physical training and
long-term return-to-work in cancer survivors. J Occup Rehabil.

[36] de Boer AG, Taskila TK, Tamminga SJ, Feuerstein M, Frings-Dresen MH, Verbeek JH (2015). Interventions to enhance return-to-work for cancer
patients. Cochrane Database Syst Rev.

