# Supplementary material for: "Rebuilding Myself"- An intervention enhancing adaptability of cancer patients to return to work: a feasibility study
Source: Rev Esc Enferm USP. 2025 May 12;59:e20240181. doi: 10.1590/1980-220X-REEUSP-2024-0181en (PMC12070807; doi:10.1590/1980-220X-REEUSP-2024-0181en)
Supplement: Translation

# Translation: “Rebuilding Myself” (Reconstruyéndome) - Una intervención que mejora la adaptabilidad de los pacientes con cáncer para reincorporarse al trabajo: un estudio de viabilidade

**DOI:** 10.1590/1980-220X-REEUSP-2024-0181es

**Authors:** Yue Shi, Yujie Guo, Jingjing Dai, Jianli Lu, Suyun Huang, Xiaopeng Lu

**Affiliations:** 1 Nantong University, School of Nursing and Rehabilitation, Nantong, China.; 2 Nantong University, Affiliated Hospital, Nantong, China.; 3 Nantong University, Affiliated Maternity and Child Health Care Hospital, Nantong, China.; 4 Yangzhou University, Affiliated Taizhou Second People’s Hospital, Taizhou, China.; 5 Nantong Sixth People’s Hospital, Nantong, China.

**Keywords:** Cáncer, Reinserción al Trabajo, Adaptabilidad, Enfermería, Estudios de Factibilidad

## Abstract

**Objetivo:**

El objetivo de esta investigación fue examinar la viabilidad y los efectos dela intervención “Rebuilding Myself” para mejorar la adaptabilidad de los pacientes con cáncer para reincorporarse al trabajo.

**Métodos:**

Se empleó un ensayo controlado aleatorio con un diseño de dos brazos yciego. El grupo de control recibió atención habitual, mientras que el grupo de intervención recibió la intervención “Rebuilding Myself”. Los efectos fueron evaluados antes de la intervención, a mitad de la intervención y después de la intervención. Los resultados incluyeron la adaptabilidad para reincorporarse al trabajo, la autoeficacia para reincorporarse al trabajo, la resiliencia mental, la calidadde vida y la capacidad para trabajar.

**Resultados:**

Los resultados mostraron una tasa de reclutamiento de 73,17%, y unatasa de retención del 80%. Se encontraron diferencias estadísticamente significativas entre los dos grupos en la adaptabilidad para reincorporarse al trabajo, la autoeficacia para reincorporarse al trabajo, la resiliencia mental, la dimensión delafunción corporal, la función emocional, la fatiga, el insomnio y la salud general delacalidad de vida.

**Conclusión:**

La intervención “Rebuilding Myself” demostró ser viable e inicialmentemejorar la adaptabilidad de los pacientes con cáncer para reincorporarse al trabajo. La intervención ayudará a proporcionar una nueva dirección para los clínicos y pacientes con cáncer para reincorporarse al trabajo.

## INTRODUCCIÓN

El cáncer se ha convertido en un importante problema de salud pública en todo el mundo^(1)^. Los estudios han demostrado que alrededor del 40% de los casos de cáncer recién diagnosticados se producen en adultos en edad laboral de entre 20 y 64 años^(2)^, y muchos de ellos son diagnosticados mientras están trabajando y posteriormente se ausentan para recibir tratamiento. Las personas que han sobrevivido al cáncer y siguen trabajando ven afectada su capacidad para conservar y avanzar en sus puestos de trabajo debido al prolongado periodo de tratamiento y sus efectos adversos^(3)^. Con los avances en las técnicas de diagnóstico y tratamiento, las tasas de supervivencia a cinco años de los pacientes con cáncer han aumentado^(4)^, lo que ha provocado un aumento constante del número de personas en recuperación. Según las proyecciones para 2020 de la Agencia Internacional para la Investigación del Cáncer (IARC), se espera que la población mundial de supervivientes de cáncer que han vivido al menos cinco años después del diagnóstico alcance los 50,6 millones^(5)^. En consecuencia, la población de supervivientes de cáncer dentro del grupo en edad laboral también está creciendo.

Uno de los principales retos para los pacientes con cáncer después del tratamiento es la reincorporación al trabajo^(6)^. Esto se refiere a la reincorporación a un empleo remunerado a tiempo completo o parcial, incluido el trabajo por cuenta propia, ya sea en el puesto original o en uno adaptado, con un promedio de horas de trabajo semanales^(7)^. La reincorporación al trabajo, como símbolo importante del resultado de la enfermedad y la reintegración en la sociedad de los pacientes con cáncer, puede reducir la carga económica de los supervivientes de cáncer^(8)^; los supervivientes de cáncer que se reincorporan al trabajo tienden a mostrar una mejor resiliencia psicológica en comparación con los que no se reincorporan a la fuerza laboral. Esta reintegración les ayuda a sentirse más conectados con la sociedad^(9)^, fomenta una sensación de «normalidad» y aumenta su autoestima^(10)^, mejorando en última instancia su calidad de vida en general^(11)^. Además, la reincorporación al trabajo aporta varios beneficios sociales, como un mayor compromiso de la fuerza laboral, una menor dependencia social y un desarrollo social más fluido. Por lo tanto, el acto de reincorporarse al trabajo es crucial no solo para el paciente individual, sino también para la sociedad en general.

Por el contrario, la situación actual de los pacientes con cáncer que se reincorporan al trabajo en China es decepcionante^(12)^. Cuando se trata de reanudar el trabajo, la mayoría de los supervivientes de cáncer se enfrentan a desafíos físicos, psicológicos y sociales. Según las investigaciones, el tratamiento a largo plazo de la enfermedad y sus diversos efectos secundarios pueden hacer que los supervivientes sufran problemas como trastorno de la imagen corporal, baja fuerza física y capacidad de trabajo reducida^(13)^; la vergüenza, la ansiedad, la depresión y otros aspectos psicológicos negativos también les dificultan la vuelta al trabajo^(14)^. Además, factores sociales como la alienación social, la discriminación laboral y la falta de apoyo de compañeros de trabajo y líderes pueden provocar desempleo, retraso en el empleo e impedimentos para la promoción^(15)^. A pesar de que la mayoría de los pacientes con cáncer sufren efectos secundarios derivados del diagnóstico y el tratamiento^(16)^, muchos están deseosos de volver al trabajo, con la excepción de los discapacitados^(17)^. La adaptación al cáncer, o ajuste psicosocial, se refiere a cómo una persona afronta los numerosos y complejos desafíos a los que se enfrenta durante la supervivencia al cáncer^(18)^. Esta adaptación se considera una medida clave de la salud general y la calidad de vida de un paciente con cáncer, y desempeña un papel crucial en su recuperación psicosocial. A los pacientes les resulta difícil adaptarse a la vuelta al trabajo después del tratamiento debido a una variedad de variables como las condiciones nacionales, las circunstancias individuales y las influencias sociales. Entre estas razones se incluyen el miedo a la recurrencia de la enfermedad, el estigma de la enfermedad, la reticencia a volver al trabajo, la baja autoeficacia y la insuficiente asistencia profesional a todos los niveles^(19)^. Las pruebas actuales^(20)^ muestran que en China faltan ensayos de intervención diseñados para ayudar a los pacientes con cáncer a adaptarse a la vuelta al trabajo. En respuesta a ello, el equipo de investigación desarrolló la intervención «Reconstruyéndome » para evaluar su viabilidad y eficacia a la hora de mejorar la adaptabilidad de los pacientes con cáncer a la vuelta al trabajo. Se planteó la hipótesis de que la intervención aumentaría la capacidad de los pacientes para volver al trabajo, mejoraría su calidad de vida y, en última instancia, contribuiría a su plena recuperación.

## MÉTODO

### Diseño y Contexto del Estudio

Este estudio se diseñó como un ensayo controlado aleatorio simple ciego de dos brazos. El protocolo de intervención se desarrolló basándose en los principios de creación y evaluación de estrategias de intervención complejas^(21)^ y siguió una secuencia de pasos que incluían investigación formativa, evaluación de viabilidad, pruebas piloto y el ensayo controlado aleatorio. El estudio de viabilidad se llevó a cabo en los departamentos y salas de oncología ambulatoria de tres hospitales de grado 3, clase A, así como en la Asociación de Rehabilitación del Cáncer de Nantong. La intervención abarcó un período de tres meses y la investigación se ajustó a las directrices del CONSORT. En la Figura 1 se puede ver un diagrama de flujo del estudio.

**Figura 1:** Flujograma del estudio.

### Participantes del Estudio

En este estudio, los participantes fueron seleccionados a través de interacciones cara a cara y recomendaciones del personal médico, y se utilizó una estrategia de muestreo de conveniencia para reclutar a los participantes que cumplían los criterios de elegibilidad. En este estudio participaron pacientes que cumplían los siguientes criterios: (1) habían recibido un diagnóstico de cáncer; (2) estaban recibiendo tratamiento rutinario en el hospital sin progresión de la enfermedad; (3) habían estado empleados anteriormente pero no habían vuelto a trabajar en el momento del estudio; (4) tenían entre 18 y 59 años (ya que 60 es la edad de jubilación en China); (5) eran capaces de leer, escribir y comprender información; y (6) eran conscientes de su diagnóstico.

Los pacientes no fueron incluidos en el estudio si tenían trastornos cognitivos o psiquiátricos, experimentaban otras afecciones de salud graves concurrentes o participaban en cualquier otro estudio relacionado.

Criterios de interrupción: Se eliminaron los pacientes que experimentaron recurrencia de la enfermedad, metástasis o empeoramiento durante la intervención. Para los pacientes que interrumpieron la intervención, los grupos de investigación tomaron las siguientes medidas: 1. Proporcionar asesoramiento psicológico a los pacientes cuya condición se deteriora con el fin de disminuir su desesperación, dolor y miedo, mientras se mantienen optimistas; 2. Alentar a los pacientes a buscar evaluación y tratamiento médico tan pronto como sea posible, y llevar a cabo revisiones periódicas; 3. Reducir el estrés proporcionándoles actividades favoritas como tocar música, ver videos, comunicarse con más frecuencia, etc.; 4. Atender las necesidades de los cuidadores, animar a los cuidadores a discutir los factores estresantes y ofrecer asistencia y apoyo positivos.

### Tamaño de la Muestra

En la actualidad, no existe una conclusión uniforme sobre el método de cálculo del tamaño de la muestra del experimento piloto por parte de los estudiosos nacionales y extranjeros. Estudios anteriores han demostrado que un tamaño de muestra de experimento piloto de 30 casos es apropiado^(22)^. Por lo tanto, se incluyeron en este estudio 30 pacientes con cáncer elegibles, con 15 casos en cada uno de los grupos de intervención y control.

## ALEATORIZACIÓN

Debido al pequeño tamaño de la muestra asignada a cada centro en este estudio, la implementación de la aleatorización en los subcentros era propensa al desequilibrio, por lo que se adoptó el método de agrupación de aleatorización central^(23)^. Debido al número de pacientes ingresados en cada centro y a la cantidad de cirugías realizadas cada año, fue difícil lograr la sincronización en la inscripción continua, por lo que se adoptó el método de inscripción competitiva para incluir a los pacientes^(24)^. El número de pacientes inscritos en cada centro no se especificó de antemano, y la inclusión y el plazo de los objetos de investigación se unificaron. Cada centro comenzó a reclutar y seleccionar pacientes al mismo tiempo para competir por la inclusión. La aleatorización se llevó a cabo utilizando números aleatorios generados por ordenador para crear una tabla de números aleatorios. Después de completar la evaluación inicial, los participantes fueron asignados aleatoriamente al grupo de intervención o al grupo de control en una proporción de 1:1. La inscripción continuó hasta que se alcanzó el número objetivo de pacientes. En este estudio, un estudiante de medicina de pregrado asignó números a los pacientes y utilizó la función «RANDBETWEEN» de Microsoft Excel para generar un conjunto de números aleatorios. Estos números se ordenaron entonces en orden ascendente. A la primera mitad de los números se les asignó un «1» y se colocaron en el grupo de intervención, mientras que a la segunda mitad se les asignó un «2» y se colocaron en el grupo de control. El estudiante escribió los números en notas adhesivas, que luego se colocaron en sobres opacos y sellados con codificación secuencial para su almacenamiento seguro. Este estudio fue realizado por un estudiante graduado en enfermería que no participó en la recopilación y el análisis de datos ni reveló la aleatorización.

## CEGAMIENTO

En la mayoría de los casos, era obvio para los pacientes si estaban en el grupo de intervención o en el grupo de control, lo que hacía imposible cegarlos a su asignación de grupo. Sin embargo, el proceso de aleatorización descrito anteriormente garantizaba que la secuencia de asignación permaneciera oculta para los investigadores involucrados en el reclutamiento de pacientes, la recolección de datos y el análisis.

## INTERVENCIONES

### Marco Teórico

Las investigaciones han indicado que las intervenciones sin un marco teórico tienden a sufrir una reducción de la calidad y la viabilidad^(25)^. Este protocolo se basó en un modelo anterior^(26)^, que ofrecía pruebas que respaldaban el enfoque de la intervención.

En la fase inicial, el equipo de investigación realizó entrevistas en profundidad con 30 pacientes con cáncer que habían regresado con éxito al trabajo, utilizando el enfoque de la teoría fundamentada. A partir de esto, desarrollaron el «Modelo de recursos de afrontamiento y experiencia de adaptación para pacientes con cáncer que regresan al trabajo»^(26)^ (Figura 2). El modelo destacó que el proceso de adaptación al regreso al trabajo para los pacientes con cáncer implicaba reconstruirse a sí mismo aprovechando los recursos disponibles. El proceso de adaptación se dividió en tres etapas: centrarse en la rehabilitación, reconstruir la autoeficacia y ajustar y planificar. El concepto central de este modelo es la reconstrucción de uno mismo.

**Figura 2:** Experiencia de adaptación al regreso al trabajo de los pacientes con cáncer y recursos para enfrentarlo (reproducido con permiso del autor JS. X).

El equipo de investigación observó que estas tres etapas estaban presentes a lo largo de todo el proceso de rehabilitación de los pacientes. La primera etapa, centrada en la rehabilitación, sirve como punto de partida para la reincorporación al trabajo, que incluye la curación, la autorreflexión, el ajuste y la mejora del aprendizaje. Reconstruir la autoeficacia es crucial para una reincorporación exitosa al trabajo, lo que implica aceptar modelos a seguir, recibir apoyo emocional y aumentar la confianza. La etapa final, de ajuste y planificación, garantiza que los pacientes puedan adaptarse con éxito a su entorno laboral buscando ayuda y estableciendo planes de carrera. Los recursos de afrontamiento se clasifican en recursos personales y externos.

Por lo tanto, basándose en el modelo teórico anterior, los grupos de investigación llevaron a cabo una revisión de la literatura y un debate en grupo para diseñar y elaborar un borrador de protocolo de intervención; seleccionaron a las partes interesadas para realizar entrevistas estructuradas con el fin de recopilar sus opiniones y revisar el borrador; y utilizaron el método Delphi para seleccionar a expertos en los campos pertinentes para llevar a cabo una consulta de expertos y, basándose en sus opiniones, revisaron y finalizaron el protocolo de intervención «Reconstruyéndome».

### Grupo de Intervención

Antes de la implementación de la intervención, se estableció el equipo de investigación, formado por un profesor de enfermería especializado en investigación de rehabilitación psicosocial de tumores, que supervisó la formación del equipo, la coordinación, la distribución de tareas y el control de calidad. Se dispuso de un asesor psicológico para proporcionar asesoramiento a los pacientes cuando fuera necesario. Cuatro estudiantes de posgrado en enfermería se encargaron de la recopilación de datos, la selección de pacientes y la ejecución de la intervención, mientras que dos estudiantes de grado en medicina se ocuparon de la generación de secuencias de asignación y el análisis de datos.

Los pacientes del grupo experimental participaron en la intervención «Reconstruyéndome », que constaba de tres temas clave y 16 cursos impartidos en persona o en línea. El primer objetivo era mejorar las habilidades de gestión de la salud de los pacientes. Esto implicaba proporcionar materiales de educación sanitaria para ayudar a los pacientes a aprender sobre la rehabilitación del cáncer, guiarlos para que reflexionaran sobre los riesgos para la salud, explorar posibles soluciones y apoyarlos en la creación y el seguimiento de planes de rehabilitación adaptados al consejo médico. El segundo objetivo era reconstruir la autoeficacia. Se animó a los pacientes a compartir sus pensamientos sobre la vuelta al trabajo, a expresar sus experiencias positivas, a establecer objetivos alcanzables y a llevar un diario para seguir su progreso, todo ello mientras reforzaban su confianza en la recuperación. Era crucial conocer cómo percibían la enfermedad y el proceso de reincorporación al trabajo la familia, los compañeros de trabajo, los supervisores, los médicos y las enfermeras del paciente, con el fin de obtener su apoyo. Para inspirar a los pacientes, se invitó a amigos que habían superado con éxito retos similares a compartir sus historias de reincorporación al trabajo. También se animó a los pacientes a modificar sus horarios de trabajo y descanso y a reanudar gradualmente las tareas laborales para recuperar la confianza. El tercer paso consistió en colaborar con profesionales médicos para crear un plan personalizado y paso a paso para la reincorporación al trabajo y el avance en sus carreras, teniendo en cuenta el estado de recuperación del paciente y las circunstancias familiares y laborales. El objetivo era crear un equilibrio entre la salud y el trabajo, al tiempo que se promovía la idea de que los pacientes buscaran ayuda de sus familiares y jefes.

Este estudio aplicó una intervención que combinaba métodos presenciales (uno a uno) y en línea (WeChat). Para el componente presencial, el proveedor y el paciente seleccionaron conjuntamente un espacio tranquilo para sus conversaciones. La intensidad de la intervención se adaptó en función de las circunstancias y necesidades individuales del paciente. Se utilizaron varios métodos, como entrevistas de comunicación uno a uno, reuniones familiares, redacción de diarios y sesiones de grupo en clases reducidas. A lo largo de la intervención, los investigadores mantuvieron el contacto con los pacientes por teléfono o WeChat para fomentar su participación continua. El formato de la intervención se basó en las preferencias de los pacientes, ajustando la intensidad de la intervención según sus condiciones. Además, los investigadores evaluaron continuamente la salud física y mental de los pacientes, lo que permitió realizar ajustes flexibles en el contenido de la intervención y ofrecer asesoramiento psicológico cuando fue necesario. Los detalles específicos de la intervención se describen a continuación (Cuadro 1).

**Cuadro 1:** Información detallada de la intervención.

Temas	Objetivos	Contenidos	Métodos
Foco en rehabilitación	1. Entender la importancia de volver a trabajar 2. Dominar el conocimiento de la rehabilitación física y mental y los métodos de autogestión 3. Implementar el plan de autogestión	1. Animar a los pacientes a expresar sus opiniones en relación a la vuelta al trabajo, comunicarse con los pacientes sobre la importancia positiva de volver al trabajo y ayudarlos a construir su confianza en su recuperación integral. 2. Realizar educación para la salud activamente, y dar panfletos educativos. Animar a los pacientes a mejorar su estudio y dominar el conocimiento de recuperación de cáncer, manteniendo una buena actitud 3. Orientar a los pacientes en la reflexión de fatores contrarios a la recuperación física y mental (malos hábitos, factores ambientales, defectos de personalidad y otros) discutiendo soluciones específicas con los pacientes, buscando apoyo de sus pares, los miembros de su familia y el personal de salud si es necesario. 4. Preguntarle a los pacientes y a sus prestadores de servicios sobre el estatus de salud y ayudarlos a desenvolver un plan de autogestión. 5. Formalizar contratos de rehabilitación con pacientes para ayudarles a aumentar su cumplimiento de la rehabilitación.	①
Reconstruir la auto-eficacia	Familiarizarse con las maneras de aumentar la auto-eficacia.	1. Comprender la decisiones de los pacientes y sus miembros de familia sus pares, líderes, colegas y staff médico en relación a la enfermedad del paciente y el regreso al trabajo; y ayudarlos a establecer una visión correcta de su rehabilitación. 2. Informar a los miembros de la familia del paciente, a sus pares, a líderes, colegas y personal médico, de la importancia de los cuidados y de apoyo que necesitan para volver al trabajo y recuperarse completamente, animándolos a ofrecer su Apoyo. 3. Entender las discusiones de los pacientes con los miembros de la familia, sus pares, los colegas, los líderes y el personal médico sobre el retorno al trabajo, preguntándole sobre sus preocupaciones y sus conflictos, discutiendo soluciones con los pacientes. 4. Animar a los pacientes a entender el apoyo que reciben de sus propias creencias, de sus miembros de la familia, de los líderes, de los colegas, de los pares, del personal de salud y otros aspectos, registrándolo en diarios y revisándolos regularmente para afirmar constantemente la fe en su recuperación integral. 5. Darle a los pacientes orientación para encontrar modelos a imitar, que hayan retornado al trabajo después del cáncer y compartir con ellos la experiencia y la energía positiva que han ganado 6. Anime a los pacientes a compartir sus experiencias superando dificultades y alcanzando el éxito en el pasado, así como los aprendizajes obtenidos de ellas. 7. Entrenamiento en autoconfianza ① Sugestión psicológica de entrenamiento: ayudar a los pacientes a sonreír a sí mismos cada día, repetir palabras positivas y darle a los pacientes afirmación y estímulo positivo en tempo. ② Entrenamiento en resiliencia mental: enseñar a los pacientes habilidades de enfrentamiento del estrés, animarlos a enfrentar las presiones activamente y guiarlos a resolver problemas, clarificando y entendiendo los problemas, quebrando los problemas complejos en pequeños pasos, proponiendo soluciones y resumiendo los problemas. ③ Los pacientes deben ser animados a gradualmente ajustar su trabajo diario y su descanso gradualmente integrándolo con el trabajo de cada día y el descanso en la etapa de trabajo. ④ Animar a los pacientes a hacer cosas relacionadas al trabajo en forma gradual.	② ①③ ④ ⑤ ①
Ajustar y planificar	Conseguir la meta de retornar gradualmente al trabajo	1. Invitar al personal de salud a hacer decisiones científicas en el momento apropiado en la posición y la carga de trabajo de los pacientes para poder retornar al trabajo de acuerdo con sus condiciones. 2. Basado en el consejo del personal de salud, preguntarle los pacientes sobre cómo es su comunicación con los miembros de su familia, con los líderes, los colegas y otros y animar al paciente a que activamente busque apoyo para retornar al trabajo si es necessário. 3. Preguntarle al paciente sobre sus objetivos de trabajo discutiendo metas apropiadas de carrera de acuerdo con su condición de recuperación haciendo un plan de carrera gradual y evaluando la relación entre salud y Trabajo. 4. Resumir los contenidos de esta intervención en un protocolo para aumentar la adaptabilidad del retorno al Trabajo.

Métodos: ①Comunicación individual y entrevista; ②Reunión de família; ③Escribir diários; ④Comunicación temática; ⑤Clases.

### Grupo de Control

Los pacientes del grupo de control solo recibieron la atención habitual, mientras que los investigadores comunicaron a los pacientes instrucciones de seguimiento personalizadas y precauciones de curación a través de WeChat. Además, los pacientes tuvieron la oportunidad de hacer preguntas al equipo de investigación sobre la rehabilitación a través de WeChat, y el equipo proporcionó respuestas detalladas y pacientes. Este enfoque permitió al equipo de investigación mantener una comunicación continua con los pacientes, fomentando la confianza y reduciendo las tasas de abandono de los pacientes. Por el contrario, los pacientes del grupo de intervención recibieron la misma atención estándar, junto con la intervención «Reconstruyéndome ».

## EVALUACIÓN DE LOS RESULTADOS

### Datos Sociodemográficos

Los siguientes datos sociodemográficos se midieron en forma de cuestionarios al inicio del estudio: sexo, edad, estado civil (casado, soltero, viudo, divorciado), educación (escuela primaria o inferior, escuela secundaria o escuela secundaria técnica, escuela secundaria superior o escuela universitaria, licenciatura, máster o superior), lugar de residencia (rural, pueblo, ciudad), creencias religiosas (sí, no), ocupación antes de la enfermedad, seguro médico, diagnóstico y estadio del cáncer, tratamiento (cirugía, radioterapia, quimioterapia, otros).

### Métricas de Viabilidad

Las métricas de viabilidad en este estudio incluyen tasas de reclutamiento, tasas de retención y satisfacción con la intervención. El método de cálculo específico es el siguiente:

(1) Tasas de reclutamiento: el número de pacientes que aceptan participar en el estudio como porcentaje del número de pacientes que cumplen los criterios de inclusión y exclusión.
(2) Tasas de retención: el número de pacientes que completan toda la intervención y rellenan el cuestionario como porcentaje del número de pacientes medidos al inicio del estudio antes de la intervención.
(3) Satisfacción con la intervención: se utiliza el método de puntuación de Likert de 5 niveles, que se divide en cinco niveles: muy insatisfecho, insatisfecho, regular, satisfecho y muy satisfecho, con puntuaciones que van del 1 al 5, respectivamente. Después de la intervención, los pacientes evaluaron todo el proceso de intervención.

### Medidas de Resultado

La principal medida de resultado fue la adaptabilidad de los pacientes con cáncer para volver al trabajo^(27)^. La escala utilizada para evaluar esto fue desarrollada por nuestro equipo de investigación y ha demostrado una gran fiabilidad y validez, con un coeficiente α de Cronbach de 0,973. Consta de 24 ítems divididos en tres categorías: enfoque de rehabilitación (6 ítems), reconstrucción de la autoeficacia (9 ítems) y ajuste y planificación (9 ítems). La escala emplea una escala Likert de 5 puntos, que va desde «totalmente en desacuerdo» hasta «totalmente de acuerdo», con puntuaciones asignadas del 1 al 5. Una puntuación global más alta refleja una mayor adaptabilidad en la capacidad de los pacientes para volver al trabajo.

Las medidas de resultado secundarias fueron la autoeficacia para volver al trabajo, la resiliencia mental, la calidad de vida y la capacidad laboral.

La versión china del Cuestionario de autoeficacia para la vuelta al trabajo^(28)^ evalúa la autoeficacia de los pacientes en su capacidad para volver al trabajo. El coeficiente α de Cronbach para esta escala oscila entre 0,90 y 0,96, lo que indica una gran fiabilidad. El cuestionario consta de 11 ítems, y los ítems 2, 6 y 9 requieren una puntuación inversa. Utiliza una escala Likert de 6 puntos, donde las respuestas van de 1 a 6 puntos. La puntuación global se calcula como la media de los 11 ítems, y una puntuación superior a 4,5 indica que el paciente tiene un mayor nivel de autoeficacia en cuanto a la reincorporación al trabajo.

La Escala de Resiliencia de Connor-Davidson^(29)^ se utilizó para evaluar la resiliencia mental de los pacientes con cáncer. Fue modificada por investigadores chinos en tres dimensiones: tenacidad (13 ítems), fuerza (8 ítems) y optimismo (4 ítems). La escala tiene un coeficiente α de Cronbach de 0,89, con una fiabilidad test-retest de 0,87. Cada elemento se califica en una escala de 0 a 5, donde 0 significa «nunca» y 5 significa «siempre». Una puntuación total más alta indica una mayor flexibilidad psicológica.

Se utilizó la versión china del Cuestionario de Calidad de Vida de la Organización Europea para la Investigación y el Tratamiento del Cáncer-Core 30^(30)^ para evaluar la calidad de vida de los pacientes con cáncer. Esta escala consta de 30 ítems, organizados en 15 dimensiones: 5 dimensiones funcionales (función física, de rol, cognitiva, emocional y social), 3 dimensiones de síntomas (fatiga, dolor, náuseas y vómitos), 1 dimensión para la salud general/calidad de vida y 6 ítems individuales (falta de aliento, insomnio, pérdida de apetito, estreñimiento, diarrea y dificultades financieras). El coeficiente α de Cronbach para cada dimensión del EORTC QLQ-C30 chino supera 0,7, excepto para la función cognitiva, y la fiabilidad test-retest está por encima de 0,73. Los ítems 29 y 30 se puntúan en una escala de 7 puntos, que va de «muy malo» a «muy bueno» (de 1 a 7 puntos), mientras que los ítems restantes utilizan una escala de 4 puntos (de 1 a 4 puntos). Las puntuaciones más altas en las dimensiones funcionales y en el estado general de salud indican una mejor calidad de vida, mientras que las puntuaciones más altas en las dimensiones de los síntomas reflejan una peor calidad de vida para los pacientes con cáncer.

Se utilizó la versión china del cuestionario del Índice de Capacidad Laboral (WAI)^(31)^ para evaluar la capacidad de una persona para realizar su trabajo. Esta escala evalúa tanto las exigencias físicas como mentales de diversas funciones, así como la salud y los recursos mentales de la persona. Consta de 7 componentes: evaluación de la capacidad laboral, necesidades, previsiones, impacto laboral, enfermedad, absentismo y estado psicológico, con una puntuación máxima de 49 puntos. Los coeficientes α de Cronbach para todas las subescalas superan 0,70, lo que indica una fiabilidad satisfactoria^(32)^. Una puntuación más alta en el cuestionario refleja una mayor capacidad para realizar tareas relacionadas con el trabajo.

### Recopilación y Análisis de Datos

Los datos para evaluar la intervención se recopilaron en tres momentos: al inicio, en la sexta semana y después de la duodécima semana, mediante un cuestionario de autocompletado. Un investigador, que no participó en la implementación ni en el análisis de la intervención, recopiló los datos. Los análisis estadísticos se realizaron con el programa SPSS versión 26.0, con un nivel de significación establecido en α = 0,05. Se utilizaron estadísticas descriptivas, como porcentajes, medias y desviaciones estándar, para resumir las variables sociodemográficas y de resultados. Para comparar las características iniciales y las medidas de resultados entre los dos grupos, se emplearon pruebas T o la prueba U de Mann-Whitney para variables continuas, y pruebas de chi-cuadrado para variables categóricas. Además, se realizaron análisis por protocolo (PP) y por intención de tratar (ITT) para evaluar la eficacia de la intervención, teniendo en cuenta los posibles abandonos y exclusiones de pacientes. En los casos de datos faltantes, se utilizaron los valores de mediciones anteriores para reemplazar los datos posteriores faltantes. Dado que el análisis ITT tiende a subestimar el efecto de la intervención, y el análisis PP puede sobreestimarlo, la fiabilidad del estudio mejora cuando ambos enfoques arrojan resultados similares^(33)^. En consecuencia, en este estudio se aplicaron ambos métodos de análisis para evaluar los resultados de la intervención.

### Consideraciones Éticas

Esta investigación recibió la aprobación ética del Comité de Ética de la Universidad de Nantong el 15 de febrero de 2019 (n.º de aprobación (2019)15) y se registró en el Registro de Ensayos Clínicos de China el 23 de marzo de 2022, con el número de registro ChiCTR2200057943.

## RESULTADOS

### Resultados del Análisis de Viabilidad

El investigador reclutó a 41 pacientes con cáncer que cumplían los criterios de inclusión y exclusión, y 30 aceptaron participar en el estudio y completaron el cuestionario de información general y el cuestionario de evaluación inicial, lo que calculó una tasa de reclutamiento del 73,17%. Durante la implementación de la intervención, 4 (13,33%) pacientes fueron excluidos debido a la recurrencia de la enfermedad, metástasis y deterioro de la enfermedad y readmitidos en el hospital para recibir tratamiento, y 2 pacientes (6,67%) se retiraron debido a un cambio en su disposición a volver al trabajo. Por lo tanto, un total de 24 pacientes en ambos grupos completaron toda la intervención y el cuestionario, lo que se calculó que da una tasa de retención del 80%. Al finalizar la intervención, 3 pacientes del grupo de intervención se sintieron «regulares», 4 «satisfechos» y 8 «muy satisfechos»; 9 pacientes del grupo de control se sintieron «generales», 5 «satisfechos» y 1 «muy satisfecho». Las puntuaciones de satisfacción de la intervención de los dos grupos de pacientes para la prueba de ji cuadrado, valor estadístico de prueba estandarizada de 2,781, P = 0,008 (P < 0,05), tienen significación estadística, lo que indica que la satisfacción de los pacientes en el grupo de intervención fue significativamente mayor que la del grupo de control. 781, P = 0,008 (P < 0,05), tienen significación estadística, lo que indica que la satisfacción de los pacientes en el grupo de intervención fue significativamente mayor que la del grupo de control.

## RESULTADOS DE LA EVALUACIÓN DE LA EFICACIA

### Comparación de los Datos de Referencia

El estado civil de los pacientes de ambos grupos era casado, y ninguno de ellos tenía creencias religiosas; el resto de las condiciones generales se analizaron mediante la prueba de chi-cuadrado, y los resultados mostraron que el valor P era superior a 0,05, y eran comparables (los resultados se detallan en la Tabla 1).

**Tabla 1:** Comparación de las condiciones generales de los dos grupos de pacientes-Nantong, China, 2022.

	Temas	Grupo Control (N = 15)	Grupo Intervención (N = 15)	*χ* ^2^	P
	≤40	3	3		
Edad	41~50	7	9	0.750	0.687
	>50	5	3		
Género	Hombre	2	2	0.000	1.000
	Mujer	13	13		
Nivel educativo	Educación Primaria o sin educación	1	1	4.400	0.355
	Primer ciclo secundario	7	7		
	Segundo ciclo secundario o técnico	4	6		
	Superior	3	0		
	Superior completo o más	0	1		
Lugar de domicilio	Interior	2	2	2.600	0.273
	Pueblo	6	10		
	Ciudad	7	3		
Seguro de Salud	Nuevo seguro Rural	1	1	1.292	0.524
	Residente urbano Seguro médico	6	9		
	Seguro del empleador	8	5		
Tipo de cáncer	Cáncer de mama	8	10	6.222	0.514
	Cáncer Cervical	2	0		
	Cáncer tiroideo	1	0		
	Cáncer del Timo	1	1		
	Cáncer de pulmón	1	1		
	Cáncer de hígado	1	0		
	Linfoma	0	2		
	Cáncer de colon	1	1		
Tipo de empleo		5	6	2.958	0.707
	Trabajador/ planta	3	2		
	Trabajador especializado	2	1		
	Personal de negocios/comercio	2	1		
	Personal de gobierno/agencias	0	2		
	Otros	3	3		
Estadio de la enfermedad	I	4	2	1.167	0.191
	II	8	8		
	III	3	1		
Tratamientos	Sólo quirúrgico	3	1	1.667	0.435
	Radiación, quimioterapia Terapia selectivas, inmunoterapia exclusivas	2	4		
	Modalidades combinadas múltiples	10	10		

### Comparación de las Puntuaciones del Índice de Evaluación de la Eficacia Entre los dos Grupos en la Línea de Base

El análisis de la línea de base de los indicadores de evaluación de la eficacia para ambos grupos de pacientes reveló un valor P superior a 0,05, que era comparable (para más detalles, véase la Tabla 2).

**Tabla 2:** Comparación de las puntuaciones del índice de evaluación de la eficacia antes de la intervención (línea de base) entre los dos grupos-Nantong, China, 2022.

Índices de evaluación		Grupo Control	Grupo Intervención	Estadísticas	*P*
Adaptabilidad a la vuelta al trabajo ( $\bar{x}$ ± s)		85.47 ± 7.62	85.87 ± 8.66	0.134	0.894
Autoeficacia para volver al trabajo ( $\bar{x}$ ± s)		4.09 ± 0.50	4.09 ± 0.46	–0.034	0.973
Resiliencia mental ( $\bar{x}$ ± s)		65.00 ± 7.97	65.47 ± 7.78	–0.162	0.872
Habilidad para trabajar ( $\bar{x}$ ± s)		28.63 ± 7.70	29.00 ± 6.15	–0.144	0.887
Calidad de vida [M(P_25_, P_75_)]	Función corporal	86.67(83.33, 93.33)	86.67(86.67, 93.33)	–0.086	0.931
	Rol funcional	83.33(75.00, 100.00)	83.33(75.00, 91.67)	–0.396	0.692
	Función emocional	83.33(70.83, 91.67)	83.33(75.00, 83.33)	–0.774	0.439
	Función cognitiva	83.33(83.33, 100.00)	83.33(83.33, 100.00)	–0.268	0.789
	Función social	66.67(66.67, 83.33)	83.33(66.67, 83.33)	–0.746	0.456
	Fatiga	77.78(66.67, 88.89)	77.78(66.67, 83.33)	–0.087	0.931
	Dolor	100.00(100.00, 100.00)	100.00(91.67, 100.00)	–0.505	0.614
	Disnea	100.00(100.00, 100.00)	100.00(100.00, 100.00)	0.000	1.000
	Insomnio	66.67(50.00, 83.33)	66.67(33.33, 100.00)	–0.242	0.809
	Pérdida de apetito	100.00(100.00, 100.00)	100.00(100.00, 100.00)	–0.544	0.586
	Náusea y vómitos	100.00(100.00, 100.00)	100.00(100.00, 100.00)	–0.949	0.343
	Constipación	100.00(100.00, 100.00)	100.00(100.00, 100.00)	–0.598	0.550
	Diarrea	100.00(100.00, 100.00)	100.00(100.00, 100.00)	0.000	1.000
	Dificultades económicas	100.00(66.67, 100.00)	100.00(66.67, 100.00)	–1.282	0.200
	Salud General	66.67(62.50, 70.83)	66.67(66.67, 66.67)	–0.345	0.730

### Comparación de las Puntuaciones del Índice de Evaluación de la Eficacia Entre los dos Grupos en el Periodo Medio (6ª Semana) y Tardío (12ª Semana)

Los resultados del estudio indicaron que en la fase media de la intervención, concretamente durante la 6ª semana, los resultados del análisis PP no revelaron diferencias estadísticamente significativas en las puntuaciones de las herramientas de evaluación entre los dos grupos de pacientes (P > 0,05) (para más detalles, véase la Tabla 3).

**Tabla 3:** Comparación de las puntuaciones del índice de evaluación de la eficacia entre los dos grupos a mitad de la intervención (a las 6 semanas de intervención) (análisis PP) - Nantong, China, 2022.

Índices de evaluación		Grupo Control	Grupo Intervención	Estadísticas	*P*
Adaptabilidad a la vuelta al trabajo ( $\bar{x}$ ± s)		86.07 ± 7.81	93.27 ± 9.38	2.048	0.054
Autoeficacia para volver al trabajo ( $\bar{x}$ ± s)		4.16 ± 0.47	4.50 ± 0.47	–1.813	0.084
Resiliencia mental ( $\bar{x}$ ± s)		65.14 ± 8.48	71.36 ± 7.83	1.902	0.070
Habilidad para trabajar ( $\bar{x}$ ± s)		29.21 ± 7.10	32.27 ± 5.13	–1.250	0.224
Calidad de vida [M(P_25_,P_75_)]	Función corporal	90.00(60.00, 100.00)	93.33(73.33, 100.00)	–1.042	0.298
	Rol funcional	83.33(50.00, 100.00)	83.33(66.67, 100.00)	–0.120	0.904
	Función emocional	83.33(66.67, 100.00)	83.33(75.00, 100.00)	–0.403	0.687
	Función cognitiva	83.33(66.67, 100.00)	100.00(66.67, 100.00)	–0.458	0.647
	Función social	83.33(66.67, 100.00)	83.33(66.67, 100.00)	–0.522	0.602
	Fatiga	77.78(55.56, 100.00)	77.78(66.67, 100.00)	–1.299	0.194
	Dolor	100.00(83.33, 100.00)	100.00(66.67, 100.00)	–0.233	0.816
	Disnea	100.00(66.67, 100.00)	100.00(66.67, 100.00)	–0.258	0.796
	Insomnio	66.67(33.33, 100.00)	66.67(33.33, 100.00)	–0.694	0.488
	Pérdida de apetito	100.00(66.67, 100.00)	100.00(66.67, 100.00)	–0.389	0.697
	Náusea y vómitos	100.00(100.00, 100.00)	100.00(83.33, 100.00)	–1.128	0.259
	Constipación	100.00(66.67, 100.00)	100.00(66.67, 100.00)	–0.389	0.697
	Diarrea	100.00(100.00, 100.00)	100.00(100.00, 100.00)	0.000	1.000
	Dificultades económicas	100.00(33.33, 100.00)	100.00(0.00, 100.00)	–0.841	0.401
	Salud general	66.67(50.00, 83.33)	75.00(66.67, 83.33)	–1.685	0.092

The study results demonstrated that at the 6th week of the intervention, which marks the middle stage, the ITT analysis indicated no statistically significant differences in the scores across the assessment tools between the two patient groups (P > 0.05) (see Tabla 4 for further details).

**Tabla 4:** Comparison of effectiveness evaluation index scores between the two groups at mid-intervention (at 6 weeks of intervention) (ITT analysis)-Nantong, China, 2022.

Índices de evaluación		Grupo Control	Grupo Intervención	Estadísticas	*P*
Adaptabilidad a la vuelta al trabajo ( $\bar{x}$ ± s)		85.67 ± 7.69	90.40 ± 10.23	1.433	0.164
Autoeficacia para volver al trabajo ( $\bar{x}$ ± s)		4.10 ± 0.51	4.33 ± 0.53	–1.179	0.248
Resiliencia mental ( $\bar{x}$ ± s)		65.13 ± 8.17	69.80 ± 7.95	1.586	0.124
Habilidad para trabajar ( $\bar{x}$ ± s)		28.37 ± 7.58	30.20 ± 7.75	–0.746	0.462
Calidad de vida [M(P_25_,P_75_)]	Función corporal	86.67(60.00, 100.00)	93.33(73.33, 100.00)	–0.763	0.445
	Rol funcional	83.33(50.00, 100.00)	83.33(66.67, 100.00)	–0.428	0.669
	Función emocional	83.33(66.67, 100.00)	83.33(75.00, 100.00)	–0.557	0.578
	Función cognitiva	83.33(66.67, 100.00)	100.00(66.67, 100.00)	–1.105	0.269
	Función social	83.33(0.00, 100.00)	83.33(33.33, 100.00)	–0.677	0.498
	Fatiga	77.78(55.56, 100.00)	77.78(66.67, 100.00)	–1.590	0.112
	Dolor	100.00(66.67, 100.00)	100.00(33.33, 100.00)	–0.105	0.916
	Disnea	100.00(66.67, 100.00)	100.00(66.67, 100.00)	–0.482	0.630
	Insomnio	66.67(33.33, 100.00)	66.67(33.33, 100.00)	–1.060	0.289
	Pérdida de apetito	100.00(33.33, 100.00)	100.00(66.67, 100.00)	–1.089	0.276
	Náusea y vómitos	100.00(66.67, 100.00)	100.00(83.33, 100.00)	–0.518	0.605
	Constipación	100.00(66.67, 100.00)	100.00(66.67, 100.00)	–0.598	0.550
	Diarrea	100.00(66.67, 100.00)	100.00(100.00, 100.00)	–1.000	0.317
	Dificultades económicas	100.00(0.00, 100.00)	100.00(0.00, 100.00)	–1.282	0.200
	Salud general	66.67(50.00, 83.33)	66.67(66.67, 83.33)	–1.705	0.088

En la semana 12 de la intervención, los resultados del análisis PP revelaron diferencias estadísticamente significativas entre los dos grupos en varias áreas, incluyendo la adaptabilidad al regreso al trabajo, la autoeficacia en el regreso al trabajo, la resiliencia mental y las dimensiones relacionadas con la función corporal, la función emocional, la fatiga, el insomnio y la salud general de la calidad de vida (P < 0,05). Sin embargo, no se encontraron diferencias significativas en las puntuaciones de los demás índices y dimensiones (P > 0,05) (véase la Tabla 5 para más detalles).

**Tabla 5:** Comparación de las puntuaciones del índice de evaluación de la eficacia entre los dos grupos en la última etapa de la intervención (a las 12 semanas de intervención) (análisis PP)-Nantong, China, 2022.

Índices de evaluación		Grupo Control	Grupo Intervención	Estadísticas	*P*
Adaptabilidad a la vuelta al trabajo ( $\bar{x}$ ± s)		86.14 ± 7.75	97.70 ± 7.79	3.592	0.002**
Autoeficacia para volver al trabajo ( $\bar{x}$ ± s)		4.16 ± 0.47	4.72 ± 0.40	–3.131	0.005**
Resiliencia mental ( $\bar{x}$ ± s)		65.29 ± 8.27	73.90 ± 8.54	2.468	0.023*
Habilidad para trabajar ( $\bar{x}$ ± s)		29.75 ± 6.67	35.00 ± 6.51	–1.927	0.068
Calidad de vida [M(P_25_,P_75_)]	Función corporal	90.00(60.00, 100.00)	100.00(73.33, 100.00)	–2.300	0.021*
	Rol funcional	91.67(66.67, 100.00)	91.67(83.33, 100.00)	–0.166	0.868
	Función emocional	83.33(66.67, 100.00)	91.67(83.33, 100.00)	–2.221	0.026*
	Función cognitiva	83.33(66.67, 100.00)	100.00(83.33, 100.00)	–0.811	0.418
	Función social	83.33(66.67, 100.00)	83.33(66.67, 100.00)	–1.354	0.176
	Fatiga	83.33(66.67, 100.00)	88.89(77.78, 100.00)	–2.303	0.021*
	Dolor	100.00(83.33, 100.00)	100.00(66.67, 100.00)	–0.244	0.807
	Disnea	100.00(66.67, 100.00)	100.00(100.00, 100.00)	–0.845	0.398
	Insomnio	66.67(33.33, 100.00)	50.00(33.33, 100.00)	–2.482	0.013*
	Pérdida de apetito	100.00(66.67, 100.00)	100.00(66.67, 100.00)	–0.845	0.398
	Náusea y vómitos	100.00(100.00, 100.00)	100.00(83.33, 100.00)	0.259	1.000
	Constipación	100.00(66.67, 100.00)	100.00(66.67, 100.00)	–1.222	0.222
	Diarrea	100.00(100.00, 100.00)	100.00(100.00, 100.00)	0.000	1.000
	Dificultades económicas	100.00(33.33, 100.00)	100.00(0.00, 100.00)	–0.426	0.670
	Salud general	66.67(50.00, 83.33)	83.33(66.67, 83.33)	–3.669	0.000***

En la semana 12 de la intervención, el análisis ITT reveló diferencias estadísticamente significativas entre los dos grupos en cuanto a la adaptabilidad de la reincorporación al trabajo, así como en las dimensiones de fatiga, insomnio y salud general dentro de la calidad de vida (P < 0,05). Sin embargo, no se observaron diferencias estadísticamente significativas para los demás indicadores y dimensiones (P > 0,05) (véase la Tabla 6 para más detalles).

**Tabla 6:** Comparación de las puntuaciones del índice de evaluación de la eficacia entre los dos grupos en la última fase de la intervención (a las 12 semanas de intervención) (análisis ITT)-Nantong, China, 2022.

Índices de evaluación		Grupo Control	Grupo Intervención	Estadísticas	*P*
Adaptabilidad a la vuelta al trabajo ( $\bar{x}$ ± s)		85.73 ± 7.63	92.73 ± 10.46	2.094	0.046*
Autoeficacia para volver al trabajo ( $\bar{x}$ ± s)		4.10 ± 0.51	4.46 ± 0.54	–1.860	0.073
Resiliencia mental ( $\bar{x}$ ± s)		65.27 ± 7.97	71.20 ± 8.65	1.953	0.061
Habilidad para trabajar ( $\bar{x}$ ± s)		28.87 ± 7.28	32.00 ± 7.17	–1.187	0.245
Calidad de vida [M(P_25_,P_75_)]	Función corporal	86.67(60.00, 100.00)	93.33(73.33, 100.00)	–1.373	0.170
	Rol funcional	83.33(50.00, 100.00)	83.33(66.67, 100.00)	–0.115	0.909
	Función emocional	83.33(66.67, 100.00)	91.67(75.00, 100.00)	–1.651	0.099
	Función cognitiva	83.33(66.67, 100.00)	100.00(83.33, 100.00)	–1.538	0.124
	Función social	83.33(0.00, 100.00)	83.33(33.33, 100.00)	–0.920	0.358
	Fatiga	77.78(55.56, 100.00)	77.78(66.67, 100.00)	–2.077	0.038*
	Dolor	100.00(66.67, 100.00)	100.00(66.67, 100.00)	–0.598	0.550
	Disnea	66.67(33.33, 100.00)	66.67(33.33, 100.00)	–2.323	0.020*
	Insomnio	100.00(33.33, 100.00)	100.00(66.67, 100.00)	–0.637	0.524
	Pérdida de apetito	100.00(66.67, 100.00)	100.00(83.33, 100.00)	–0.048	0.962
	Náusea y vómitos	100.00(66.67, 100.00)	100.00(66.67, 100.00)	–1.439	0.150
	Constipación	100.00(66.67, 100.00)	100.00(100.00, 100.00)	–1.000	0.317
	Diarrea	100.00(0.00, 100.00)	100.00(0.00, 100.00)	–1.282	0.200
	Dificultades económicas	66.67(50.00, 83.33)	66.67(66.67, 83.33)	–2.975	0.003**
	Salud general	77.78(55.56, 100.00)	77.78(66.67, 100.00)	–2.077	0.038*

PS: *P < 0.05

**P < 0.01

***P < 0.001

## DISCUSIÓN

El protocolo de intervención se basó en el modelo teórico elaborado por nuestro equipo y, a través de la revisión de la literatura, el debate en grupo, las entrevistas estructuradas y la consulta a expertos de Delphi, se construyó el protocolo para comprender plenamente las sugerencias de diferentes grupos de personas, como los pacientes con cáncer y sus familias, el personal clínico y los expertos en diversos campos. El protocolo de intervención tuvo en cuenta las diferencias individualizadas de los pacientes con cáncer y adoptó intervenciones presenciales (uno a uno) y en línea (WeChat) que se complementan entre sí; al mismo tiempo, desarrolló intervenciones personalizadas basadas en las propias condiciones y necesidades de los pacientes sin interferir en su vida diaria ni aumentar su carga.

Los resultados del estudio de viabilidad revelaron una tasa de reclutamiento de la intervención del 73,17%, lo que demuestra que la mayoría de los pacientes con cáncer en remisión tenían necesidades relacionadas con la intervención, estaban dispuestos a participar en este estudio de intervención y estaban ansiosos por adaptarse a la vuelta al trabajo. Los estudios han demostrado que la recomendación del personal médico es un método eficaz de reclutamiento^(34)^, y 21 pacientes (70%) fueron reclutados utilizando este método en este estudio, lo que promueve el desarrollo de una relación de confianza entre el ejecutor de la intervención y el paciente y mejora la adherencia a la intervención. En segundo lugar, los resultados revelaron que los pacientes del grupo de intervención estaban sustancialmente más satisfechos con la intervención que el grupo de control, lo que indica que los pacientes con cáncer estaban dispuestos a aceptarla. Además, la tasa de retención de la intervención en este estudio fue del 80%, con 4 (13,33%) pacientes que abandonaron debido a la recurrencia de la enfermedad, metástasis y empeoramiento de la readmisión en el hospital para recibir tratamiento, y 2 pacientes (6,67%) que se retiraron del estudio debido a un cambio en su disposición a volver al trabajo, lo que indica que la adherencia de los participantes a la intervención en este estudio fue buena. Además, los resultados del ensayo controlado aleatorio indicaron que el protocolo puede mejorar la adaptabilidad de los pacientes para volver al trabajo, la autoeficacia para volver al trabajo, la resiliencia mental y la dimensión de la función corporal, la función emocional, la fatiga, el insomnio y la salud general de la calidad de vida, lo que ha mostrado efectos preliminares y proporciona una base práctica para futuros ensayos clínicos con muestras más grandes. En resumen, el protocolo de intervención es factible.

Un estudio^(35)^ destacó que el proceso de reincorporación al trabajo de los pacientes con cáncer está influenciado por múltiples factores y requiere un enfoque integrado que combine métodos psicológicos, vocacionales y físicos. En una revisión sistemática de Cochrane^(36)^, los autores clasificaron en cuatro tipos las intervenciones destinadas a mejorar la adaptabilidad de los pacientes con cáncer en su reincorporación al trabajo: física, psicológica, ocupacional y multidisciplinaria, proporcionando definiciones para cada categoría. Este protocolo incorpora métodos físicos, psicológicos y ocupacionales para ofrecer apoyo de rehabilitación multidisciplinar a los pacientes, con el objetivo de mejorar su capacidad para volver al trabajo como parte de una estrategia de rehabilitación integral. Por lo tanto, este protocolo de investigación demuestra su aplicabilidad práctica.

Este estudio llevó a cabo un ensayo controlado aleatorio para evaluar la adaptabilidad de los pacientes chinos con cáncer en su regreso al trabajo. Desde una perspectiva de innovación teórica, el estudio anterior de la teoría fundamentada del equipo de investigación, basado en fuentes creíbles, desarrolló un modelo teórico para comprender las experiencias de los pacientes con cáncer y los recursos de afrontamiento relacionados con el regreso al trabajo, e introdujo el nuevo concepto de «adaptabilidad al regreso al trabajo». El equipo también propuso el protocolo de intervención adaptativa «Reconstruyéndome » para pacientes con cáncer, que incorpora diversas estrategias de intervención. En términos de innovación práctica, los resultados del estudio podrían motivar a futuros investigadores clínicos a realizar ensayos controlados aleatorios multicéntricos a gran escala. Los protocolos de intervención validados pueden ayudar a los pacientes con cáncer a reanudar su trabajo y lograr la recuperación psicosocial, proporcionando una base para futuras investigaciones y nuevas direcciones en el campo de los estudios de reincorporación al trabajo.

## LIMITACIONES DE LA INVESTIGACIÓN

Este estudio sin embargo, presentaba algunas deficiencias. Los resultados del estudio de viabilidad mostraron que la ventaja de esta intervención para mejorar la capacidad de trabajo de los pacientes no era evidente, lo que podría estar relacionado con el menor tiempo de intervención del estudio; los resultados de los análisis PP e ITT fueron parcialmente inconsistentes, y la credibilidad de los resultados del estudio debe mejorarse. Además, el tamaño de la muestra incluida en el estudio fue pequeño y los resultados fueron más inestables; en el futuro, el tamaño de la muestra puede ampliarse aún más y el número de visitas de seguimiento puede incrementarse para explorar más a fondo los efectos a largo plazo de este protocolo.

## CONCLUSIÓN

Este estudio introdujo por primera vez la intervención «Reconstruyéndome» para la recuperación física y mental de los pacientes con cáncer. El protocolo de intervención demostró su viabilidad y mostró mejoras iniciales en la adaptabilidad de los pacientes con cáncer para volver al trabajo, la autoeficacia para volver al trabajo, la resiliencia mental y la calidad de vida en general. El protocolo fue de gran valor para la realización gradual de la rehabilitación física, psicológica y social integral de los pacientes con cáncer. También tiene el potencial de guiar el desarrollo de futuros ensayos controlados aleatorios y proporcionar nuevas ideas para los pacientes que deseen volver al trabajo.
